# HIRA-SETDB1-H3K9me3 axis regulates chromatin architecture in leukemia cells

**DOI:** 10.1016/j.jbc.2026.113081

**Published:** 2026-04-27

**Authors:** Mayur Balkrishna Shirude, Anjali Devarajan, Sai Adarsh Sahu, Ananda Mukherjee, Debasree Dutta

**Affiliations:** 1 BRIC-Rajiv Gandhi Centre for Biotechnology, Regenerative Biology Program, Thiruvananthapuram, Kerala, India; 2 Manipal Academy of Higher Education, Manipal, Karnataka, India; 3 Regional Centre for Biotechnology, Faridabad, Haryana, India; 4 Cancer Biology Laboratory, Department of Medical Oncology, Sri Ramachandra Institute of Higher Education and Research, Chennai, India

**Keywords:** BCR-ABL, chromatin compaction, chronic myeloid leukemia, EZH2, histone chaperone, HIRA, HP1α, H3K9me3

## Abstract

Histone cell cycle regulator A (HIRA) confers chromatin accessibility and regulates developmental hematopoiesis. Previously, we showed that HIRA expression is higher in patient samples from chronic myeloid leukemia (CML) compared to samples from healthy individuals. However, the underlying mechanism that connects HIRA with chromatin reorganization and pathogenesis of leukemia associated with abnormal hematopoiesis remains unexplained. We developed a HIRA-knockdown K562 CML cell line model for this study, as this cell line showed a maximal expression of HIRA in the myeloid lineage. A proteome-wide analysis demonstrated the association of HIRA with components of chromatin organization in K562 cells. Fluorescence Recovery After Photobleaching and fluorescence lifetime imaging microscopy-forster resonance rnergy transfer microscopy and molecular interaction studies revealed increased chromatin compaction and altered spatial distribution of chromatin towards the nuclear periphery upon downregulation of HIRA in K562 cells. Mechanistically, enhanced chromatin compaction was attributed to increased histone H3K9me3 and HP1α levels mediated by histone methyltransferase SET Domain Bifurcated Histone Lysine Methyltransferase 1 (SETDB1). The enrichment of histone H3.3 and the reduction in H3K27me3 levels, resulting from the loss of enzyme unit Enhancer of Zeste Homolog 2 recruitment at the *SETDB1* and *HP1α* promoters in HIRA-knockdown cells, led to an increase in their expression. This HIRA-SETDB1-H3K9me3 axis contributed to restricted cell proliferation along with loss in expression of the Breakpoint Cluster Region protein- Abelson Tyrosine-Protein Kinase 1 fusion protein that causes CML. Thus, loss of HIRA promotes global chromatin condensation and redistribution, thereby regulating the Breakpoint Cluster Region protein- Abelson Tyrosine-Protein Kinase 1 expression and cell proliferation. Our findings highlight how elevated HIRA expression contributes to the pathogenesis of CML and establish a regulatory axis that could be further explored for therapeutic interventions.

The changes in chromatin architecture are a dynamic process that dictates cell fate during differentiation. Embryonic and hematopoietic stem cells differentiation process is accompanied by dramatic global chromatin architectural changes (1). Differentiation of hematopoietic stem cells to blood cells show progressive chromatin condensation mediated by H3K9 methylation that gets redistributed towards nuclear periphery (2). Previous report from our lab demonstrated the role of histone chaperone Histone cell cycle regulator A (HIRA) in regulation of RUNX1 indispensable for hemogenic to hematopoietic transition (3). HIRA, a replication independent histone chaperone associates with UBN1, CABIN1 and transiently with ASF1A to form a complex that incorporates H3.3 and marks active transcription (4, 5, 6). H3.3 exhibits apparently contradicting roles at different genetic loci (7). HIRA has been shown to regulate chromatin accessibility and modulate the process of developmental hematopoiesis (8). But, whether HIRA could play a similar role in regulation of the chromatin accessibility in leukemia, remain unexplored. Leukemia is a cancer of the blood and bone marrow. Different types of leukemia are being reported depending on the type of blood cell getting affected. Our earlier study demonstrated an induced expression of HIRA in chronic myeloid leukemia (CML) patient samples and in K562 cell line derived from CML patient at blast phase (9). The presence of fusion protein arising from chromosomal translocation of t(9;22) (q34;q11.2), Breakpoint Cluster Region protein- Abelson Tyrosine-Protein Kinase 1 (BCR-ABL) oncoprotein (10), results in constitutive active tyrosine kinase activity (11) that influences cell proliferation, apoptosis and survival among others (12). Starting with an asymptomatic chronic phase, it goes through accelerated phase and finally reaches the blast phase. Aberrant or uncontrollable proliferation and impaired differentiation marks leukemia and our earlier study demonstrated the role of HIRA in regulation of proliferation *versus* differentiation of CML cells (9). Loss in HIRA level resulted in inhibition of proliferation while induction of differentiation to megakaryocyte fate (9). But how HIRA could modulate the chromatin architecture leading to a regulatory role in leukemia remain unexplored till date. Although a significant number of studies have been conducted in understanding the role of HIRA in developmental hematopoiesis, but, its role in leukemia have not been explored further. Chromatin architecture aberrations are linked to various human diseases, including cancer (13). The induced expression of HIRA in CML patient samples and in K562 cell line and its regulatory role in chromatin states prompted us to investigate the hypothesis that HIRA may regulate global chromatin architecture, thereby influencing the cellular fate of the CML cells.

## Results

### HIRA associates with chromatin organization in K562, CML cell line

**Figure 1:**
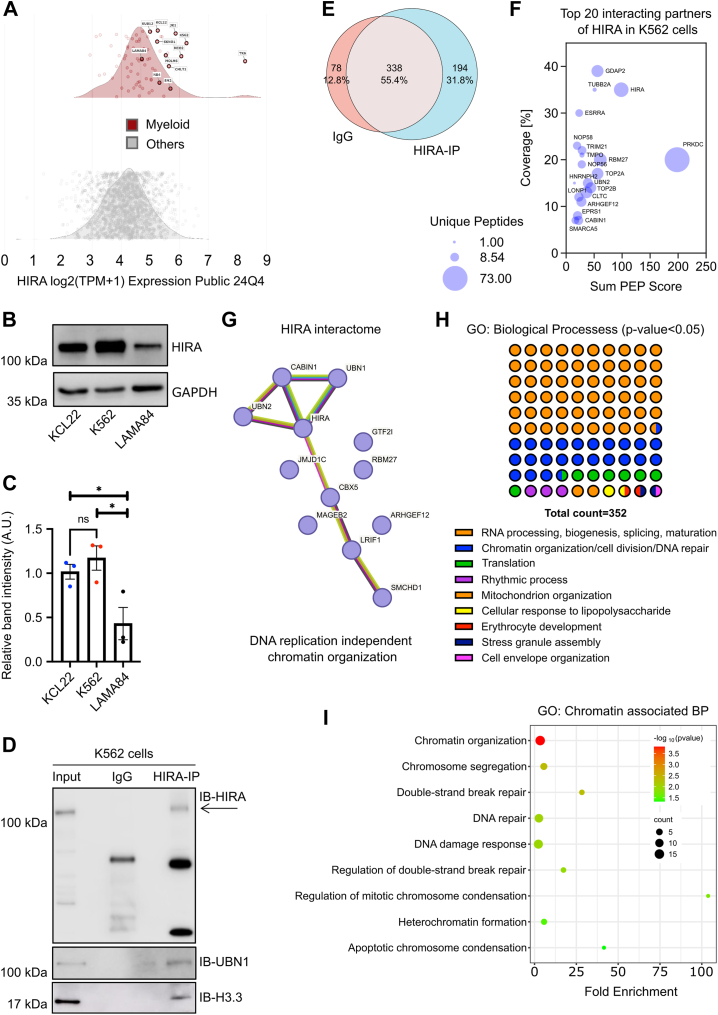
**HIRA associates with chromatin organization in K562, CML cell line.***A*, the density plots illustrate the relative expressions of mRNA and protein for HIRA in K562 cells. The *upper panel* depicts the distribution of HIRA transcript and protein levels within the myeloid lineage, whereas the *lower panel* presents the same distribution for other lineages. A two-class comparison was made by finding the q-value and a moderated estimate of the difference between the myeloid lineage and all other cell lines' means to account for differences in HIRA expression. This was performed using a custom analysis method in the DepMap portal. *B*, Western blot analysis for the expression of HIRA in K562, LAMA84 and KCL22 cells, thereby confirming the RNA expression analyzed in *A*. *C*, bar graph represents band intensity of HIRA normalized to GAPDH in different CML cell cells. Unpaired *t* test was performed for the statistical analysis, ∗*p* < 0.05, ns = not significant, n = 3 biological replicates. *D*, Western blot analysis for the confirmation of immunoprecipitation of K562 cell lysate with HIRA. Interaction of histone UBN1 and H3.3 with HIRA. *E*, HIRA-IP sample along with IgG control was subjected to LC-MS/MS analysis for the detection of interacting partners of HIRA. Venn diagram demonstrate 194 partners unique to HIRA-IP sample. *F*, a visual representation of the top 20 HIRA interactive partners based on the Sum PEP score, percent coverage, and unique peptides. Sum PEP Score is sum of negative logarithms of PEP values for all peptide spectrum matches. The higher the value, the higher the credibility. peptide spectrum matches indicate the total number of identified peptide sequences matched for the protein. Coverage displays the percentage of the protein sequences covered by identified peptides. The bubble plot was generated using a multiple variable analysis in GraphPad Prism 10. *G,* STRING database analysis for the interaction partner of HIRA. The cluster for DNA replication independent chromatin assembly shows interaction of HIRA with its complex proteins. *H*, Gene ontology analysis by Database for Annotation Visualization and Integrated Discovery bioinformatics tool (https://david.ncifcrf.gov) for the enriched chromatin associated biological processes (BP) for HIRA deduced from LC-MS/MS study. Graph represents all significant biological processes. Each circle represents one biological process. *I*, bubble graph demonstrate the gene ontology analysis by Database for Annotation Visualization and Integrated Discovery bioinformatics tool for the enriched chromatin associated biological processes for HIRA. HIRA, histone cell cycle regulator A; CML, chronic myeloid leukemia; PEP, posterior error probability; GO, gene ontology.

Being the chaperone that deposits histone H3.3, HIRA regulates the chromatin, thereby influencing key cellular and molecular events. A DepMap custom analysis function indicated most of the cell lines within the myeloid lineage which clustered on the right-hand side of the curve are blast-phase CML in origin, implying the fact that CML cell lines might associate with higher levels of HIRA (Fig. 1*A*). Interestingly, CML cell line, K562 showed one of the highest *HIRA* mRNA expressions among them, except for the TK6 cell that was at the far-right side of the plot, indicating a possible outlier. The LAMA84 CML cell line exhibited reduced HIRA level relative to both K562 and other CML cells (Fig. 1*A*). Western blot analysis for the expression of HIRA in K562, LAMA84 and KCL22 (with intermediate expression of HIRA as DepMap data, Fig. 1*A*) demonstrated significant reduction in HIRA in LAMA84 cells in comparison to K562 and KCl22 cells whereas no significant difference in HIRA level was observed between K562 and KCL22 (Fig. 1, *A–C*). This result validated the DepMap data and corroborated our earlier finding (9). Therefore, we used the K562 cell line for further molecular and functional studies. Our objective was to investigate the functional role of HIRA in the modulation of chromatin architecture, which may contribute to a regulatory role in leukemia. However, before proceeding, it was essential to ascertain whether HIRA is linked to chromatin organization in CML cells. Hence, we determined the HIRA interactome in K562 cells.

Whole cell protein lysate from K562 cells were subjected to immunoprecipitation with HIRA followed by LC-MS/MS analysis. The resulting HIRA-pulled down lysate was confirmed with presence of HIRA, histone variant H3.3 and UBN1, the *bona fide* HIRA interacting partners (Fig. 1*D*). A total of 194 hits were obtained for HIRA after deducting the ones present in IgG sample (Fig. 1*E*) (Tables S6–S8 sheet 2). The results showed an increase in enrichment (based on Sum posterior error probability score, percentage coverage and unique peptide attributes) of known HIRA complex partner UBN2, CABIN1 and chromatin remodeler SMARCA5 among top 20 interaction partners (Fig. 1*F*). STRING database analysis demonstrated the presence of seven major clusters (Fig. S1*A*) including rRNA modifications, RNA splicing, chromosome segregation/cell division/mitotic spindle, metabolic processes, cytoplasmic translation and DNA replication independent chromatin assembly (Fig. 1*G*). The functional annotation analysis using Database for Annotation Visualization and Integrated Discovery bioinformatics tool, demonstrated significant enrichment of three major clusters and six minor clusters (Fig. 1*H*) (Table S8-sheet 1). Chromatin organization cluster appeared among the top 15 significant biological processes enriched (Fig. S1*B*, Fig. 1*I*), including chromosome segregation, DNA repair, mitotic chromosome condensation (Fig. 1*I*) along with other processes. Hence, association of HIRA with different proteins of chromatin organization intrigued us in investigating the role of increased expression of HIRA in CML cells in regulating the chromatin architecture.

### HIRA regulates chromatin compaction

**Figure 2:**
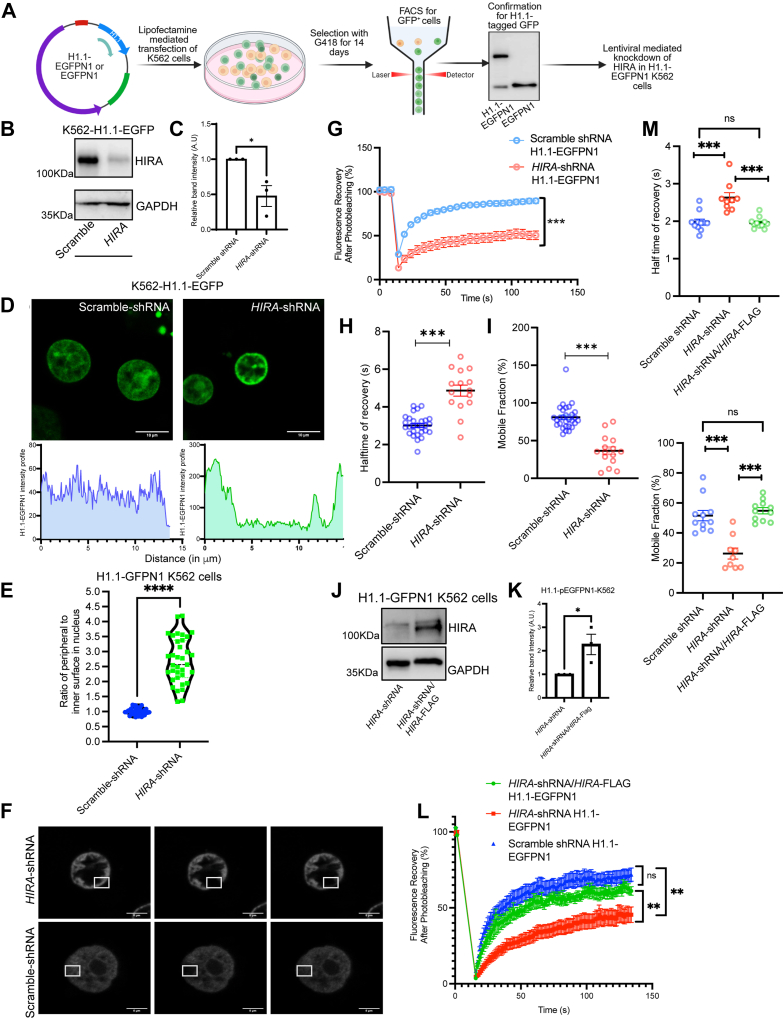
**HIRA regulates chromatin compaction in CML cells.***A*, schematic representation of the generation of H1.1-EGFPN1 expressing K562 cells harboring either scramble shRNA or *HIRA*-shRNA. *B*, Western blot analysis for the expression of HIRA in H1.1-EGFPN1 positive scramble and *HIRA*-shRNA expressing K562 cells. Lentiviral particles harbouring *HIRA*-shRNA or scramble shRNA were transduced in K562 cells, followed by selection in presence of puromycin. Stable HIRA-downregulated K562 cell lines were generated. These cells were further processed for the expression of H1.1-EGFPN1 as mentioned in A. *C*, bar graph represents band intensity for the knockdown of HIRA in H1.1-EGFPN1 K562 cells normalized to GAPDH. Unpaired *t* test was performed for the statistical analysis, ∗*p* < 0.05, N = 3 biological replicates. *D*, live cell imaging for the expression of EGFP in the same set of cells analyzed in *B*. *Lower panel* demonstrate the intensity curve of GFP across the nucleus. *E, violin* plot represents the distribution of ratio of H1.1-GFP intensity at the periphery to the inner surface within the nucleus of cells expressing scramble-shRNA or *HIRA*-shRNA in K562 cells. Each dot represents a cell. Mann-Whitney U test was performed for the statistical analysis, ∗∗∗∗*p* < 0.0001, N = 35 cells. *F*, Fluorescence Recovery After Photobleaching analysis in K562 cells. The micrographs represent Region of Interest (*square box*) for pre-bleach, bleach and post-bleach area. *G*, fluorescence recovery curve of the same set of cells analyzed in *F*. Unpaired *t* test statistical analysis was performed, ∗∗∗*p* < 0.001. *H* and *I*, scatter plots represent the half time for recovery of fluorescence and mobile fractions in scramble and *HIRA*-shRNA expressing K562 cells. Two-tailed unpaired *t* test was performed for the statistical analysis, ∗∗∗*p* < 0.001. *J*, Western blot analysis for the expression of HIRA in *HIRA*-shRNA K562 cells rescued with expression of *HIRA*-Flag expression. *K*, bar graph represents band intensity for the induction of HIRA in *HIRA*-shRNA -H1.1-EGFPN1 K562 cells normalized to GAPDH. Unpaired *t* test was performed for the statistical analysis, ∗*p* < 0.05, N = 3 technical replicates. *L*, Fluorescence Recovery After Photobleaching analysis was conducted upon rescue of *HIRA*-shRNA with ectopic expression of *HIRA*-FLAG in the same cells. Fluorescence recovery after photobleacing demonstrates a similar pattern in rescued cells and scramble-shRNA expressing cells, while significant difference of the recovery pattern with *HIRA*-shRNA expressing cells. *M* and *N*, bar graphs demonstrate the half-time for the recovery of GFP and mobile fraction in rescued cells in comparison to scramble and *HIRA*-shRNA expressing cells. Two-tailed unpaired *t* test was performed for statistical analysis. ∗∗∗∗*p* < 0.0001. HIRA, histone cell cycle regulator A; CML, chronic myeloid leukemia.

Chromatin is highly dynamic, undergoing compaction and decompaction to regulate DNA replication, transcription, and damage repair. To understand the role of HIRA in modulating chromatin architecture in CML cells, we compared the global euchromatin and heterochromatin content of the wild type and HIRA depleted K562 cells using Fluorescence Recovery After Photobleaching (FRAP). Histone H1.1 being a linker histone is mobile in loosely compacted chromatin (*i.e.,* euchromatin) while its mobility gets restricted in a densely packed or compacted chromatin (*i.e.,* heterochromatin) (14). Hence, we tagged GFP at C-terminus of H1.1 for FRAP analysis (Fig. 2*A*). The GFP-positive cells were sorted by Fluorescence-activated cell sorting (FACS) (Fig. 2*A*, Fig. S1, *A–C*). Stable K562 cells expressing H1.1-EGFPN1 were generated followed by downregulation of HIRA by lentiviral mediated shRNA expression (Fig. 2, *A–C*). Interestingly live cell imaging of the scramble K562 cells showed uniformly distributed H1.1-EGFPN1 within the nucleus, on the contrary HIRA knockdown K562 cells showed H1.1-EGFPN1 significantly localized at the nuclear periphery (Fig. 2, *D* and *E* Additional Videos 1 and 2). FRAP analysis demonstrated that the Region of Interest (ROI) bleached either at the nuclear periphery or in the middle of the nucleus showed up to 80% recovery in scramble-shRNA cells indicating the euchromatin state while cells expressing *HIRA*-shRNA could recover only up to 40% of the fluorescence (Fig. 2, *F* and *G*). Striking difference in the recovery rate of H1.1-EGFPN1 indicated that down-regulation of HIRA resulted in an increased heterochromatin content accompanied by its re-distribution towards the nuclear periphery (Fig. 2, *D–G*). Significant increase in the half time for fluorescence recovery after photobleaching was observed in cells transfected with *HIRA*-shRNA compared to scramble-shRNA (Fig. 2*H*). On the contrary, a significant decrease in mobile fraction was observed in the *HIRA*-shRNA expressing cells in comparison to the scramble-shRNA expressing cells (Fig. 2*I*). Interestingly, upon rescue of the *HIRA*-shRNA cells with ectopic expression of *HIRA*-FLAG (Fig. 2, *J* and *K*), we observed a significant increase in the recovery of the fluorescence, increase in the mobile fraction and reduction in the half time of recovery (Fig. 2, *L–N*). This substantiated the fact that loss in expression of HIRA indeed contributes to the compaction of the chromatin in K562 cells.

**Figure 3:**
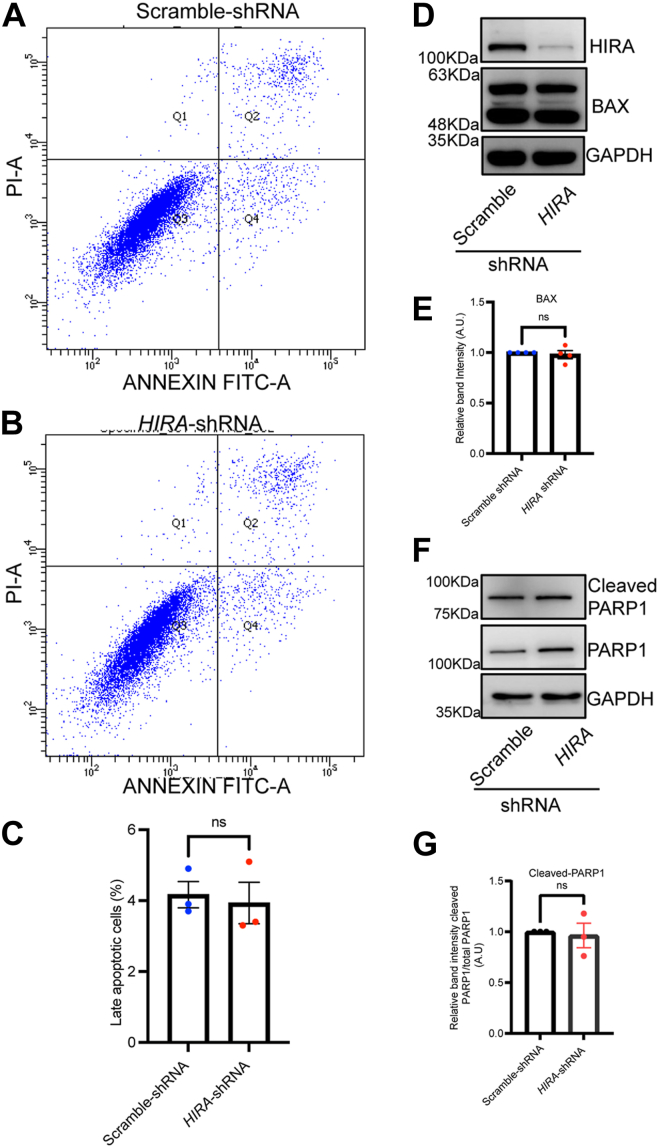
**Loss in HIRA does not induce apoptosis.***A* and *B*, scramble shRNA and *HIRA*-shRNA expressing K562 cells were stained with Annexin-V–FITC and propidium iodide, followed by fluorescence-activated cell sorting analysis. Q2 represents FITC/PI-labeled late apoptotic cells. *C*, graph represents the fraction of apoptotic cells in the same set of cells analyzed in *A* and *B*. Unpaired *t* test was performed for the statistical analysis, ns = not significant, N = 3 biological replicates. *D*, Western blot analysis for the expression of apoptosis associated marker BAX in stably transduced scramble-shRNA and *HIRA*-shRNA K562 cells. *E*, bar graph represents band intensity for the expression of BAX normalized to GAPDH level in cells analyzed in *D*. Unpaired *t* test was performed for the statistical analysis, ns = not significant, N = 3 biological replicates. *F*, Western blot analysis for the expression of apoptosis associated marker cleaved PARP1 in comparison to PARP1 in scramble-shRNA and *HIRA*-shRNA expressing K562 cells. *G*, bar graph represents band intensity for the expression of cleaved PARP1 with respect to corresponding PARP1 level normalized to GAPDH level in cells analyzed in *F*. Unpaired *t* test was performed for the statistical analysis, ns = not significant, N = 3 biological replicates. HIRA, histone cell cycle regulator *A*.

Multiple studies have implicated compaction of chromatin as one of the hallmark features of apoptosis (15, 16, 17). Hence, we investigated whether this chromatin compaction associated with loss in HIRA could induce apoptosis. Annexin-V staining of K562 cells expressing *HIRA*-shRNA failed to demonstrate any significant change in the percentage of Annexin-V positive cells in comparison to K562 cells expressing scramble shRNA (Fig. 3, *A–C*). Cleaved PARP1 (18) and BAX (19) are bonafide markers of apoptosis. We observed that downregulation of HIRA failed to induce any alteration in the level of cleaved BAX (Fig. 3, *D* and *E*) and PARP1 level (Fig. 3, *F* and *G*).

Next, we were intrigued to determine whether altered level of HIRA in CML cells could result in the spatial redistribution of chromatin.

### HIRA facilitates spatial distribution of chromatin

**Figure 4:**
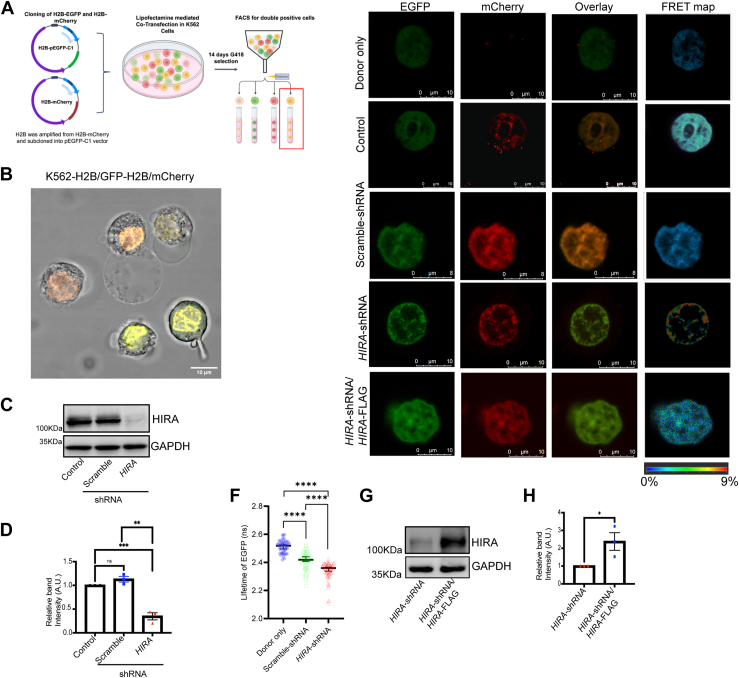
**HIRA regulate spatial distribution of the chromatin in CML cells.***A*, schematic representation of generation of double positive (H2B-pEGFPC1 and H2B-mCherry) K562 cells harboring either scramble or *HIRA*-shRNA. These cells stably expressed the scramble-shRNA or the *HIRA*-shRNA. *B*, live cell imaging for the co-expression of GFP and mCherry in K562 cells. *C*, Western blot analysis for the expression of HIRA in double positive control, scramble and *HIRA*-shRNA expressing K562 cells. *D*, bar graph represents the band intensity for the knockdown of HIRA normalized to GAPDH in double positive K562 cells. Unpaired *t* test was performed for the statistical analysis, ∗*p* < 0.05, ∗∗<*p* < 0.01, N = 3. *E*, live cell images for the donor-acceptor and FRET efficiency map in K562 cells expressing different set of constructs. *F*, scatter plot demonstrates the significant reduction in life-time of GFP in the same set of cells analyzed in *C*. Two-tailed unpaired *t* test was performed for the statistical analysis, ∗∗∗*p* < 0.001. *G*, Western blot analysis for the expression of HIRA in *HIRA*-shRNA and *HIRA*-shRNA/*HIRA*-Flag expressing K562 cells. *H*, bar graph represents the band intensity for the rescue of HIRA normalized to GAPDH in double positive K562 cells. Unpaired *t* test was performed for the statistical analysis, ∗*p* < 0.05, N = 3. HIRA, histone cell cycle regulator A; CML, chronic myeloid leukemia.

FRAP analysis gave an insight to the role of HIRA in modulating chromatin architecture. In order to quantify the chromatin compaction induced by depletion of HIRA and visualize the spatial distribution across the nucleus, we employed Fluorescence lifetime imaging microscopy (FLIM)-Forster Resonance Energy Transfer (FRET). H2B tagged with donor (EGFP) and acceptor (mCherry) fluorophore separately were stably transfected in K562 cells followed by the sorting of Two fluorophore positive cells (2FP) cells (Figs. S1 and S2 These cells were subjected to the knockdown of HIRA using lentiviral mediated shRNA expression (Fig. 4, *A–D*). Fluorescence lifetime imaging microscopy-forster resonance energy transfer (FLIM-FRET) allows to distinguish the euchromatin and heterochromatin based on the lifetime of the donor fluorophore H2B-EGFP. H2B-EGFP and H2B-mcherry has been previously used as a FRET pair for FLIM-FRET analysis (20). When chromatin is compacted H2B-EGFP (Donor fluorophore) and H2B-mCherry (acceptor fluorophore) being in close proximity, lead to FRET, consequently reducing the lifetime of the fluorophore. Due to the difference in compaction, heterochromatin will show increased FRET efficiency while euchromatin will show less FRET efficiency. Pixel wise counting of the donor lifetime allows calculating FRET efficiency, which represents the state of chromatin. Upon down-regulation of HIRA in 2FP cells, a re-organization of chromatin was observed (Fig. 4*E*). FRET efficiency map of HIRA down-regulated cells showed similar observation from FRAP with spatial redistribution of the chromatin at the nuclear periphery and increase in compaction or enhanced presence of heterochromatin (Fig. 4*E*). Loss in HIRA led to significant decrease in lifetime of the EGFP in comparison to the control cells (Fig. 4*F*). Upon rescue of the HIRA level by ectopic expression of HIRA-FLAG in the 2FP cells (Fig. 4*G*), the FRET-efficiency was reduced with redistribution of the chromatin throughout the nucleus (Fig. 4*E*, lowermost panel). FLIM-FRET analysis demonstrated that HIRA downregulation leads to increase in the heterochromatin content associated with alteration in the spatial distribution of the chromatin.

### Downregulation of HIRA induces a global increase in the H3K9me3 level

**Figure 5:**
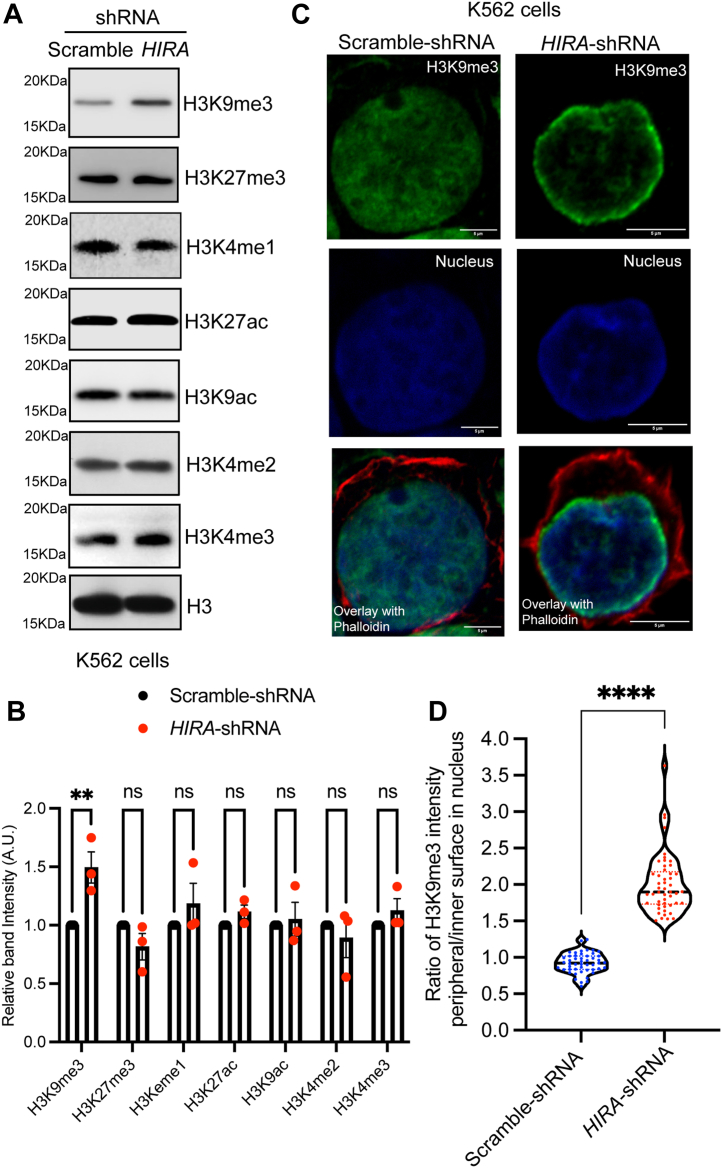
**Loss in HIRA induce global increase in H3K9me3 level.***A*, Western blot analysis for the expression of different histone modification marks in stably transduced scramble *versus HIRA*-shRNA expressing K562 cells. *B*, bar graph representing the band intensities of histone modification marks normalized to total histone H3 level. A two-way ANOVA statistical analysis was done followed by Šídák’s multiple comparisons tests, ∗∗*p* < 0.01, ns = not significant, n = 3 biological replicates. *C*, immunofluorescence analysis for the expression of H3K9me3 in scramble *versus HIRA*-shRNA expressing K562 cells. *D, violin plot* represents the distribution of ratio of fluorescence intensity of H3K9me3 modification present at the periphery to the inner surface within the nucleus of cells expressing scramble-shRNA or *HIRA*-shRNA in K562 cells. Each *dot* represents a cell. Mann-Whitney U test was performed for the statistical analysis, ∗∗∗∗*p* < 0.0001, N = 50 cells. HIRA, histone cell cycle regulator A.

The histone variant H3.3 is a confirmed interacting partner of HIRA, and this association persists in K562 cells (Fig. 1*D*). Here, we observed an increase in heterochromatin upon downregulation of HIRA. Hence, we investigated into the global change in histone modification patterns that could result in increased heterochromatin formation. We determined the level of histone H3K4me3, H3K4me2, H3K4me1, H3K4ac, H3K9ac, H3K27me3, H3K9me3 levels in response to the loss in expression of HIRA in K562 cells (Fig. 5*A*). Interestingly, among all of them, a significant increase was observed in the level of *bona fide* heterochromatin mark, H3K9me3 only (Fig. 5, *A* and *B*). Immunofluorescence analysis indicated the presence of H3K9me3 at the nuclear periphery in HIRA-knockdown cells (Fig. 5*C*). A significant enrichment in the peripheral localization of H3K9me3 was observed in *HIRA*-downregulated cells in comparison to cells expressing scramble-shRNA (Fig. 5*D*). Earlier, a similar spatial distribution pattern of H1.1-EGFP expressing chromatin were observed upon downregulation of HIRA in K562 cells (Figs 2 and 3). Since, HIRA is responsible for the recruitment of histone H3.3 at active enhancers or promoters within the genome, we investigated into any alteration in global localization of H3.3 upon downregulation of HIRA. No significant change in the distribution pattern of H3.3 was observed in the K562 cells expressing scramble or *HIRA*-shRNA (Fig. S3). Thus, it’s the increase in the level of H3K9me3 that might contribute to the enhanced compaction of the chromatin or its spatial distribution near the nuclear periphery.

But how is H3K9me3 altered upon loss in HIRA?

### SET Domain Bifurcated Histone Lysine Methyltransferase 1 facilitates chromatin remodeling by enhanced H3K9me3 level in HIRA-depleted K562 cells

**Figure 6:**
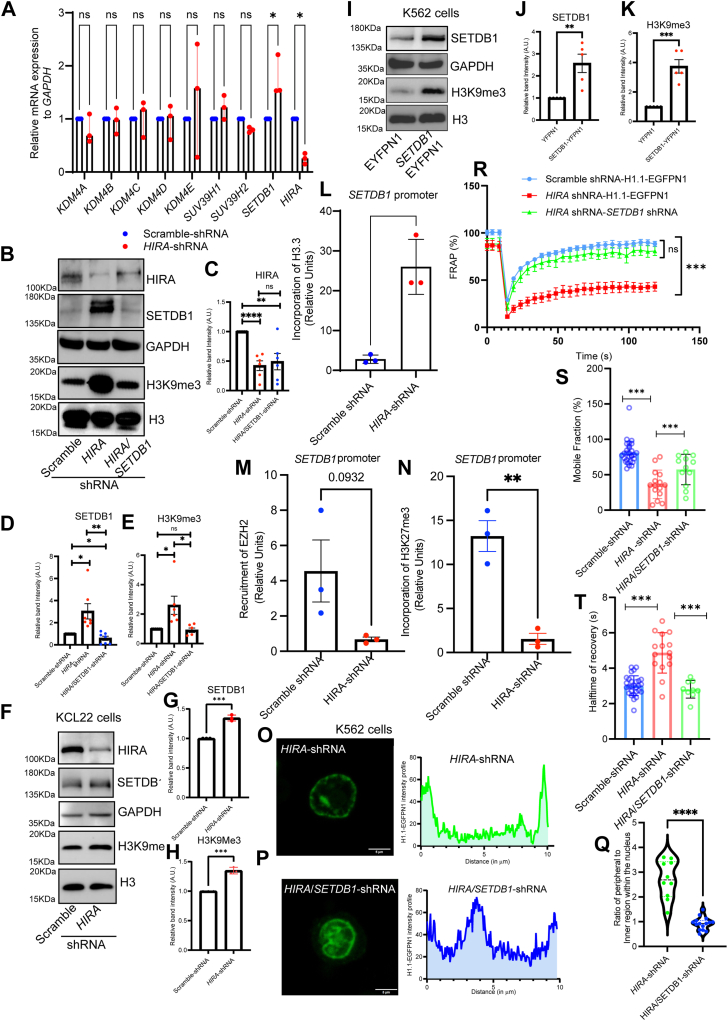
**HIRA-SETDB1-H3K9me3 axis regulates chromatin architecture in CML cells.***A*, quantitative RT-PCR analysis for the expression of different histone methyltransferases and demethylases in stably transduced scramble *versus HIRA*-shRNA expressing K562 cells. Unpaired *t* test was performed for the statistical analysis, ∗*p* < 0.05, ns = not significant, N = 3. *B*, Western blot analysis for the expression of SETDB1, HIRA, H3K9me3 level in scramble *versus HIRA*-shRNA and stably transduced *HIRA/SETDB1* shRNA expressing K562 cells. *C–E*, bar graph representing the band intensities of HIRA, SETDB1 and H3K9me3 respectively, normalized to GAPDH. Unpaired *t* test was performed for the statistical analysis, ∗*p* < 0.05, ∗∗*p* < 0.01, ∗∗∗*p* < 0.001, ns = not significant, N = 3. *F*, HIRA-SETDB1-H3K9me3 axis in additional CML cell line, KCL22. HIRA expression was downregulated in KCL22 cells by lentiviral mediated *HIRA*-shRNA expression and the samples were analyzed for the expression of HIRA, SETDB1 and H3K9me3 by Western blot analysis. *G* and *H*, bar graph representing the band intensities of SETDB1 and H3K9me3 normalized to GAPDH respectively. Unpaired *t* test was performed for the statistical analysis, ∗∗∗*p* < 0.001, N = 3 biological replicates. *I*, YFP tagged SETDB1 was ectopically stably expressed in K562 cells and the resulting samples were analyzed for the expression of SETDB1 and H3K9me3 by Western blot analysis. *J* and *K*, bar graph representing the band intensities of SETDB1 and H3K9me3 normalized to GAPDH respectively. Unpaired *t* test was performed for the statistical analysis, ∗∗∗*p* < 0.001, ns = not significant, N = 3. *L*, bar graph represents the quantitative ChIP analysis for the recruitment of histone variant H3.3 within *SETDB1* promoter in scramble *versus HIRA*-shRNA expressing K562 cells. Two-tailed unpaired *t* test was performed for the statistical analysis, ∗∗*p* < 0.01, N = 3. *M* and *N*, bar graph represents the quantitative ChIP analysis for the recruitment of enzyme unit Enhancer of Zeste Homolog 2 and incorporation of H3K27me3 within *SETDB1* promoter in scramble *versus HIRA*-shRNA expressing K562 cells. Two-tailed unpaired *t* test was performed for the statistical analysis, ∗∗*p* < 0.01, N = 3. *O* and *P*, live cell imaging for the expression of GFP in the *HIRA*-shRNA and *HIRA*/*SETDB1*-shRNA expressing K562 H1.1-EGFPN1 cells. Lentiviral particles expressing *SETDB1* shRNA were transduced in K562 H1.1-EGFPN1 cells harboring *HIRA*-shRNA, mentioned in Figure 2. Intensity curve of GFP across the nucleus has been shown. *Q*, *violin plot* demonstrates the ratio of intensity of H1.1 at the periphery *versus* inner surface of nucleus of cells analyzed in *O* and *P*. Each dot represents a cell. Mann-Whitney U test was performed for the statistical analysis, ∗∗∗∗*p* < 0.0001, N = 10 cells for *HIRA*-shRNA K562 cells; N = 15 for *HIRA/SETDB1*-shRNA K562 cells. *R*, fluorescence recovery curve of *HIRA*-shRNA and *HIRA*/*SETDB1*-shRNA expressing K562 H1.1-EGFPN1 cells. Unpaired *t* test statistical analysis was performed, ∗∗∗*p* < 0.001. *S* and *T*, scatter plots represent the mobile fractions and half time for fluorescence recovery in scramble, *HIRA*-shRNA and *HIRA*/*SETDB1*-shRNA expressing cells. Two-tailed unpaired *t* test was performed for the statistical analysis, ∗∗∗*p* < 0.001, N = 3. HIRA, histone cell cycle regulator A; CML, chronic myeloid leukemia.

The addition or removal of functional group from the lysine residue of the histone tails are accomplished by a set of histone modifying enzymes, the demethylase or the methyltransferases. Hence, to investigate upon the fact that which mechanism drive the increase in H3K9me3 level, we determined the level of these modifying enzymes. Interestingly, among the demethylases, we failed to observe any significant change upon loss of HIRA expression (Figs. 6*A*, S4), however, a significant increase in SET Domain Bifurcated Histone Lysine Methyltransferase 1 (SETDB1) level was observed both at the mRNA and protein level (Figs. 6, *A–D*, S4*A*). But does this increase in SETDB1 expression contribute to the increase in H3K9me3 level? To answer that, we knocked down SETDB1 in *HIRA*-knockdown K562 cells (Fig. S4*B*) and observed the level of H3K9me3. The increase in H3K9me3 level was abrogated with loss in SETDB1 level (Fig. 6, *B–E*). Thus, SETDB1 mediate the increase in H3K9me3 level in the context of loss of HIRA in K562 cells. Does this axis of HIRA-SETDB1-H3K9me3 exist in other CML cell lines? Hence, we explored the KCL22 cell line as the level of HIRA was comparable to K562 (Fig. 1, *A* and *B*). Upon downregulation of HIRA in KCL22, a significant increase in SETDB1 and H3K9me3 level was observed in comparison to cells expressing scramble shRNA (Fig. 6, *F–H*). Next, we asked whether the increase in SETDB1 expression could lead to the increase in global level of H3K9me3 in CML cells. SETDB1 was ectopically expressed in K562 cells and Western blot analysis demonstrated a significant increase in H3K9me3 level in these cells compared to cells expressing control empty vector (Fig. 6, *I–K*).

But, how does loss in HIRA level enhance SETDB1 expression? Incorporation of H3.3 mediated by HIRA complex and/or DAXX/ATRX within the chromatin, dictate contradictory functions in the context of development and differentiation (7). We determined the incorporation or loss of H3.3 in the scramble and *HIRA*-shRNA expressing K562 cells within the *SETDB1* promoter. Interestingly, we observed a significant enrichment in the incorporation of H3.3 within the *SETDB1* promoter upon downregulation of HIRA (Fig. 6*L*). However, in coherence with our earlier study (9), significant loss in H3.3 incorporation was observed at the *RUNX1* promoter while no significant difference was observed at the *GAPDH* promoter (Fig. S4, *C* and *D*). Recent report indicated that depletion of HIRA resulted in de-repression of genes that were repressed by Polycomb Repressor Complex 2 (PRC2) in hematopoietic stem cells (21). Interestingly, *SETDB1* is also a target gene for PRC2 enzyme unit Enhancer of Zeste Homolog 2 (EZH2) (22). No significant change in expression of EZH2 was observed in K562 cells expressing *HIRA*-shRNA in comparison to control cells (Fig. S4, *E–G*). However, upon knockdown of HIRA, a significant loss in recruitment of EZH2 was observed within the *SETDB1* promoter with subsequent loss in the repressive H3K27me3 mark (Fig. 6, *M* and *N*). No loss/gain in recruitment of EZH2 was observed within *GAPDH* intergenic region (Fig. S4*H*). This finding underlies the enhanced expression of SETDB1 in K562 cells expressing *HIRA*-shRNA. The K562 cells generated for FRAP analysis (Fig. 2) were further manipulated for the downregulation of SETDB1, leading to the generation of *HIRA*/*SETDB1*-H1.1-EGFPN1 double knockdown cells. Live cell imaging of HIRA-downregulated cells demonstrated similar chromatin architecture with enhanced presence in nuclear periphery (Fig. 6*O*) as mentioned in Figure 2 whereas upon downregulation of SETDB1, these cells demonstrated the chromatin distribution throughout the nucleus, similar to cells expressing only scramble-shRNA (Fig. 6*P*). A ratio of fluorescence intensity of H1.1 at peripheral to inner surface within the nucleus demonstrated significant loss in the double-knockdown K562 cells in comparison to the *HIRA*-shRNA cells (Fig. 6*Q*). FRAP analysis indicated increase in recovery of fluorescence upon loss in SETDB1 expression in HIRA-downregulated cells (Fig. 6*R*). Similarly, an increase in mobile fraction and decrease in halftime in recovery was observed in the HIRA/SETDB1 knocked down cells (Fig. 6, *S* and *T*). Thus, we provide evidence that the SETDB1 expression was responsible for the enhanced H3K9me3 level upon loss in HIRA that aid in the regulation of chromatin architecture of CML cells.

Subsequently, we investigated the changes that leukemia cells undergo due to chromatin compaction. Our earlier study showed that downregulation of HIRA reduced K562 cell proliferation; thus, we sought to determine whether this axis is involved in regulating the cell proliferation.

### Chromatin compaction mediated by loss in HIRA restricts proliferation of K562 cells

**Figure 7:**
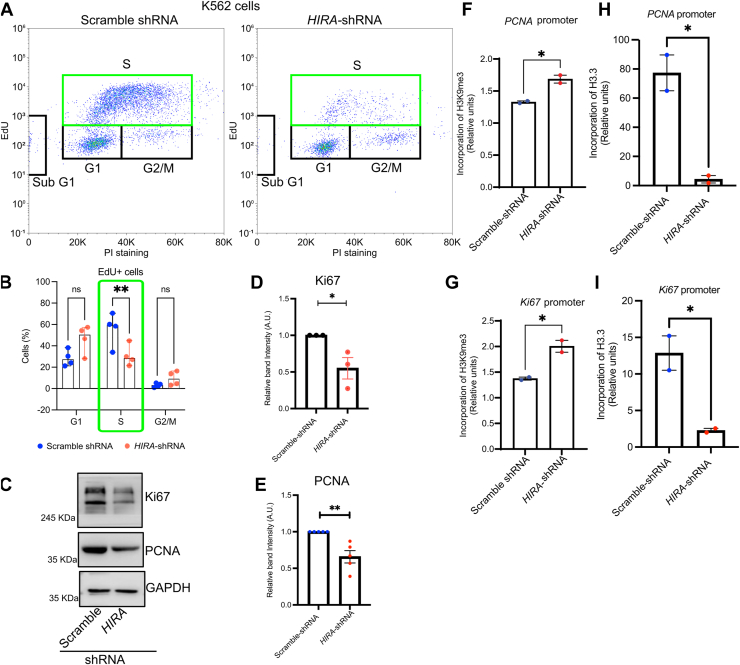
**Loss in HIRA-mediated chromatin compaction restricts proliferation of K562 cells.***A* and *B*, flow Cytommetry analysis for the quantification of EdU positive population in stably transduced K562 cells expressing scramble and *HIRA*-shRNA. The bar graph illustrates the percentage of cells in various phases of the cell-cycle, determined by PI staining for the total DNA content and EdU incorporation with S phase indicated by a *green rectangle*. Two-way ANOVA with Sidak correction for *p*-value analysis was performed. The *error bar* represent median with 95% CI for four biological replicates. ∗∗*p* < 0.01, N = 4. *C*, Western blot analysis for the expression of Ki67 and PCNA in scramble and *HIRA*-shRNA expressing K562 cells. *D* and *E*, bar graphs represent the band intensity of Ki67 and PCNA normalized to GAPDH in the samples analyzed in *C*. Unpaired *t* test was performed for the statistical analysis, ∗∗*p* < 0.01, N = 3. *F* and *G*, bar graphs represent the quantitative ChIP analysis for the incorporation of histone H3K9me3 mark within the promoter region of Ki67 and PCNA. Two-tailed unpaired *t* test was performed for the statistical analysis, ∗*p* < 0.05, N = 2. *H* and *I*, quantitative ChIP analysis for the incorporation of histone H3.3 within *Ki67* and *PCNA* promoters on K562 cells expressing scramble-shRNA and *HIRA*-shRNA. Two-tailed unpaired *t* test was performed for the statistical analysis, ∗*p* < 0.05, N = 2. HIRA, histone cell cycle regulator A.

Here, we performed EdU incorporation assay to further confirm the role of HIRA in proliferation of CML cells. Upon downregulation of HIRA, we observed a significant reduction in EdU incorporation in K562 cells (Fig. 7, *A* and *B*). A significant reduction in EdU positive cells were observed in the S-phase cell cycle upon loss in HIRA expression in K562 cells (Fig. 7*B*). No significant enrichment in sub-G1 phase cell population was observed upon downregulation of HIRA (Fig. S5*A*). Further, CYCLIN E1 protein levels remained unchanged in HIRA-depleted K562 cells, indicating that the loss of HIRA does not induce blockage at G1 phase (Fig. S5*B*). This further consolidates our earlier finding that loss in HIRA inhibit proliferation. But does loss in HIRA influence the expression of proteins associated with cell proliferation by altering the chromatin state at the respective loci? How does HIRA mediate it? To demonstrate this, at first we detected the protein level of the proliferation markers, PCNA and Ki67 in the K562 cells having scramble or *HIRA*-shRNA. Upon downregulation of HIRA, a significant decrease in both Ki67 and PCNA was observed (Fig.e 7, *C–E*). Does this loss in expression mediate from a compacted chromatin? Quantitative ChIP analysis demonstrated an increased enrichment of heterochromatin mark H3K9me3 within the respective promoters of *PCNA* and *Ki67* in HIRA downregulated cells in comparison to control cells (Fig. 7, *F* and *G*). No significant difference in H3K9me3 incorporation was observed within the *GAPDH* promoter (Fig. S5*C*). Additionally, and quite expectedly, significant loss in H3.3 incorporation was observed within *PCNA* and *Ki67* promoters, upon loss in HIRA (Fig. 7, *H* and *I*).

**Figure 8:**
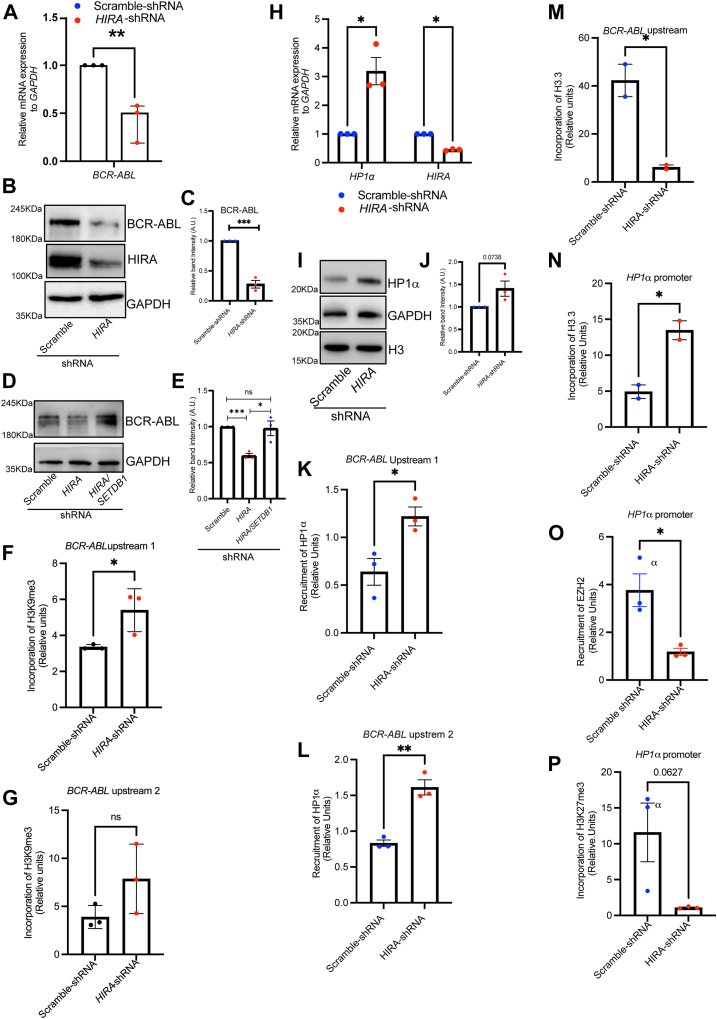
**HIRA regulate BCR-ABL expression in K562 cells.***A*, quantitative RT-PCR for the expression of BCR-ABL in scramble and *HIRA*-shRNA expressing K562 cells. Unpaired *t* test was performed for the statistical analysis, ∗∗∗*p* < 0.01, N = 3. *B*, Western blot analysis for the expression of HIRA and fusion BCR-ABL in the same set of cells analyzed in *H*, N = 3. *C*, bar graph represents the band intensity of BCR-ABL normalized to GAPDH in samples analyzed in *A*. Unpaired *t* test was performed for the statistical analysis, ∗∗∗*p* < 0.001, N = 3. *D*, Western blot analysis for the expression of BCR-ABL expression in cells analyzed in Figure 6*B*. *E*, bar graph represent the band intensity of BCR-ABL normalized to GAPDH. Unpaired *t* test was performed for the statistical analysis, ∗∗∗*p* < 0.001, N = 3. *F* and *G*, bar graphs represent the qChIP analysis for the incorporation of H3K9me3 mark within the upstream/promoter region of BCR locus. Unpaired *t* test was performed for the statistical analysis, ∗*p* < 0.05, ns = not significant, N = 3. *H*, quantitative RT-PCR for the expression of HP1α in scramble and *HIRA*-shRNA expressing K562 cells. Unpaired *t* test was performed for the statistical analysis, ∗*p* < 0.05, N = 3. *I*, Western blot analysis for the expression of HP1α in samples analyzed in *H*. *J*, bar graphs represent the band intensity of BCR-ABL normalized to GAPDH. Unpaired *t* test was performed for the statistical analysis, N = 3. *K* and *L*, bar graphs represent the qChIP analysis for the recruitment of HP1α within the upstream/promoter region of *BCR* locus. Unpaired *t* test was performed for the statistical analysis, ∗*p* < 0.05, N = 3. *M*, bar graphs represent the qChIP analysis for the incorporation of H3.3 within the upstream region of BCR locus. Unpaired *t* test was performed for the statistical analysis, ∗*p* < 0.05, N = 2. *N*, bar graphs represent the qChIP analysis for the incorporation of H3.3 within the *HP1α* promoter region. Unpaired *t* test was performed for the statistical analysis, ∗*p* < 0.05, N = 2. *O*, bar graphs represent the qChIP analysis for the recruitment of enzyme unit Enhancer of Zeste Homolog 2 within the *HP1α* promoter region. Unpaired *t* test was performed for the statistical analysis, ∗*p* < 0.05, N = 3. *P,* bar graphs represent the qChIP analysis for the incorporation of H3K27me3 within the *HP1α* promoter region. Unpaired *t* test was performed for the statistical analysis, N = 3. BCR-ABL, Breakpoint Cluster Region protein- Abelson Tyrosine-Protein Kinase 1; HIRA, histone cell cycle regulator A.

Earlier studies have implicated that BCR-ABL activates multiple downstream pathways, resulting in enhanced proliferation, decreased apoptosis, aberrant adhesion and migration, and genetic instability in CML cells (23). The expression of BCR-ABL is essential for the maintenance and also for the progression of this disease (23). We reasoned that reduced proliferation might result from loss in BCR-ABL level. Interestingly, downregulation of HIRA resulted in loss in expression of BCR-ABL both at the mRNA and protein level (Fig. 8, *A–C*). On the contrary, the expression of BCR-ABL was rescued upon expression of *SETDB1*-shRNA in HIRA-downregulated K562 cells (Fig. 8, *D* and *E*). Expression of the fusion protein BCR-ABL is guided by the *BCR* promoter. Quantitative ChIP analysis demonstrated a significant enrichment of H3K9me3 mark within the upstream sequence one of the *BCR* locus upon downregulation of HIRA in K562 cells (Fig. 8*F*). A similar enrichment of H3K9me3 was observed in the upstream sequence 2, but the difference was not statistically significant (Fig. 8*G*). It is well evidenced that H3K9me3 serves as the docking place for binding of HP1 isoforms, resulting in chromatin compaction (24). Interestingly, loss in HIRA induced the expression of HP1α both at the protein and mRNA level (Fig. 8, *H–J*). Quantitative ChIP analysis demonstrated significant increase in the recruitment of HP1α at the *BCR-ABL* promoter regions (Fig. 8, *K* and *L*) while no significant difference was observed within *GAPDH* promoter (Fig. S5*D*). Expectedly, loss in H3.3 incorporation was observed within the *BCR-ABL* promoter upon downregulation of HIRA (Fig. 8*M*). But, how do the level of HP1α gets enhanced upon loss in HIRA? We determined the incorporation of H3.3 followed by recruitment of EZH2/H3K27me3 within the *HP1α* promoter. ChIP-seq data demonstrated HP1α to be a target gene for EZH2 (21). A significant enhancement in H3.3 level was demonstrated within the HP1α promoter upon downregulation of HIRA (Fig. 8*N*) whereas loss in the recruitment of EZH2 and H3K27me3 level was observed (Fig. 8, *O* and *P*). Thus, a similar mechanism underlies the regulation of SETDB1 and HP1α in K562 cells upon downregulation of HIRA.

**Figure 9:**
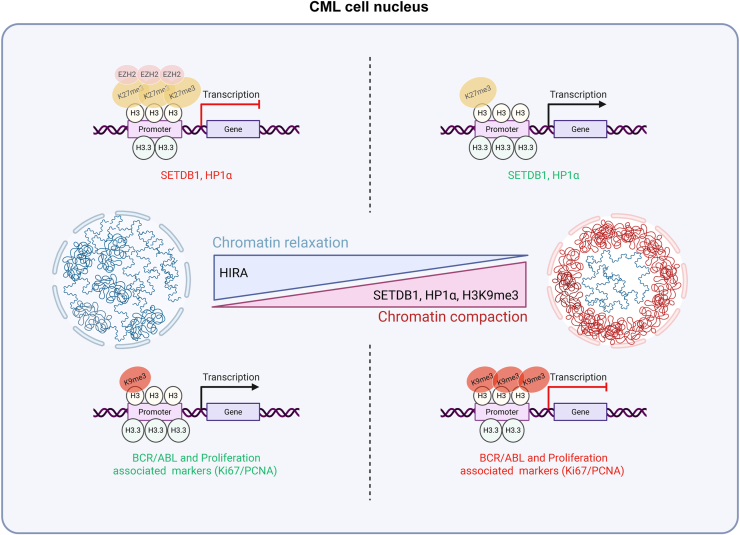
**Regulation of chromatin architecture by histone chaperone HIRA in leukemia.** Proposed model depicting the regulation of chromatin architecture in CML cells by HIRA. The model was generated using Biorender. HIRA, histone cell cycle regulator A; CML, chronic myeloid leukemia.

Here in this study, we provide experimental evidence to show that histone chaperone HIRA could regulate the expression of histone methyl transferase SETDB1, thereby regulating the chromatin architecture in terms of compaction and spatial distribution through the incorporation of heterochromatin mark H3K9me3, which in turn could regulate proliferation of the CML cells by regulating the expression of BCR-ABL fusion protein (Fig. 9).

## Discussions

Chronic myeloid leukemia, a hematopoietic stem cell disease, progresses from the chronic phase through the accelerated phase to the blast phase. The chimeric BCR-ABL protein is a tyrosine kinase (TK) that is constitutively active and activates a variety of signaling pathways including PI3K, MAPK, JAK/STAT, regulating cellular functions like proliferation, apoptosis among others and collectively result in the malignant transformation (11, 12, 23). With the advent of BCR-ABL TK inhibitors, the treatment of CML was completely transformed. Nevertheless, resistance to these TKIs and the increased cost of these drugs along with adverse effects of long-term usage, the prognosis of blast-phase CML, and treatment-free remission provide contemporary problems in the domain of CML (25). BCR-ABL is essential and adequate for the onset and sustenance of CML. Hence, it would be beneficial to explore a mechanism that could lead to reduced expression of BCR-ABL. Our present study provides experimental evidence that downregulation of HIRA could lead to loss in BCR-ABL in K562 cells.

Earlier reports provided evidence on role of HIRA in the formation of senescence-associated heterochromatin foci (24, 26). But how it is associated with leukemia has not been studied. HIRA selectively incorporates histone variant H3.3 in a replication independent manner into the nucleosomes within defined chromatin domains. But the incorporation of H3.3 has contradictory roles at different loci. In fact, the H3.3-HIRA complex is responsible for the formation of heterochromatin in senescent cells (27). We observed that upon loss in HIRA, chromatin compaction and spatial distribution was altered in the CML cells. But the spatial redistribution of the chromatin at the nuclear periphery indicated an additional factor and that is H3K9me3 deposition, which is a *bona fide* heterochromatin mark.

We concur that our findings of HIRA inhibit chromatin compaction may seem to be at odds with the enrichment of heterochromatin-associated proteins within the HIRA interactome. In order to better reconcile this seeming contradiction, we have now broadened the conversation. In particular, we suggest that HIRA may play a regulatory or antagonistic role rather than a cooperative one in its interactions with heterochromatin-related proteins and other repressive chromatin regulators. In fact, according to recent research, depending on the genomic context, incorporation of H3.3 by HIRA may be able to counteract the formation of heterochromatin (7, 8). In our investigation, we demonstrated that upregulation of SETDB1, a known H3K9 methyltransferase, mediates the increased H3K9me3 levels and chromatin condensation caused by loss of HIRA. This suggests that HIRA might typically work to restrict SETDB1 expression, which would stop heterochromatin from spreading. Its location at the interface between euchromatin and heterochromatin, where it plays a protective architectural role, may therefore account for the observed enrichment of heterochromatin-associated proteins in the HIRA interactome. While our study does not directly test this model, this interpretation is consistent with prior observations of HIRA’s context-specific role in regulating chromatin architecture (7, 8, 28).

As mentioned earlier, the incorporation of H3.3 or H3K9me3 is context specific, and it is also known that depletion of HIRA might increase H3.3 pool leading to chromatin rearrangement (7). We anticipate that wherever we visualize a relaxation of the chromatin, other factor including incorporation of H3.3 might be responsible for the chromatin state which demands further experimental evidence.

An ambiguity which is very much pertaining to this study is why the recruitment of H3.3 was enriched upon loss in HIRA within different promoters. For that, we investigated the various components of HIRA complex and other histone chaperones associated with H3.3 recruitment. No significant alteration in expression of UBN1, ASF1A, CABIN1, ASF1B or H3.1 chaperone CAF1 was observed upon loss in HIRA (Fig. S6, *A–H*). However, DAXX and ATRX, the other H3.3 chaperone demonstrated significant increase in the expression at the RNA level. It is well documented that both these chaperone complexes of HIRA/CABIN1/UBN1/2 or DAXX/ATRX can compensate for one another (7, 29, 30). Additionally, incorporation of H3.3 within heterochromatin regions have been more associated with the DAXX/ATRX complex. We predict that a compensation mechanism mediated by the DAXX/ATRX complex might be responsible for the increase in H3.3 incorporation within genes associated with heterochromatin formation. However, this understanding requires additional experimental evidence, especially in a *HIRA*-knockout model to negate any contribution of HIRA in the incorporation of H3.3.

SETDB1 associated increase in H3K9me3 level upon HIRA downregulation point to another new finding. Recent report indicated that SETDB1 negatively regulate pro-leukemia genes and thereby suppress AML mediated by H3K9me3 (31). Our present study also indicates a similar outcome. Interestingly, TCGA database analysis demonstrated significant negative correlation with SETDB1 in AML patient samples [Acute Myeloid Leukemia (OHSU, Cancer Cell 2022), 942 samples] (Fig. S7). Interestingly, our LC-MS/MS analysis also demonstrated the interaction of HIRA with H3K9me3 demethylase JMJD1C (Fig. 1*E*). It could be another side of the story wherein loss in HIRA might result in loss in the recruitment of JMJD1C thereby resulting in compaction of the chromatin. Additional experimental evidence would provide further proof to this hypothesis.

Although, we demonstrated the differential incorporation of H3.3 within the *SETDB1* promoter resulted in upregulation of SETDB1 expression thereby inducing the level of H3K9me3, but there are already studies conducted which provide ample evidence that HIRA itself can in fact regulate histone modification, as demonstrated in yeast (28). So, we predict that other additional factors might be involved in this regulatory axis responsible for this chromatin status.

The level of HIRA in CML cell lines are comparatively higher than other myeloid cancer cell lines (Fig. 1*A*), however, LAMA84 which is also a CML cell line, express significantly reduced HIRA level (Fig. 1, *A–C*). But our mechanistic data generated in two cell lines of K562 and KCL22 indicate that the axis of HIRA-SETDB1-H3K9me3 could be a general mechanism of chromatin compaction in CML cell lines. It is plausible to hypothesize that elevated HIRA levels result in increased BCR-ABL expression, potentially contributing to the drug resistance observed in CML patients (32).

We predict that a similar phenomenon might exist in cancers of other origins that demonstrated increased expression of HIRA as per the DepMap portal derived mRNA analysis in other non-myeloid cell lines. So, it would be interesting to explore whether the HIRA-SETDB1-H3K9me3 axis is a conserved phenomenon or the one specific to only CML.

The major outcome of this study provides a mechanistic foundation for future studies investigating how chromatin architectural changes contribute to gene regulation and leukemia cell proliferation.

## Conclusions

Our findings uncover a novel epigenetic regulatory axis wherein histone chaperone HIRA restricts chromatin compaction and maintains spatial distribution of chromatin by limiting SETDB1-mediated H3K9 trimethylation. Thus, elevated HIRA expression in CML cells induces BCR-ABL levels through this axis, thereby contributing to its pathogenesis. Loss of HIRA leads to increased heterochromatin content, peripheral reorganization of heterochromatin consequently limiting cellular proliferation. These results position HIRA as a key architectural regulator of chromatin in CML cells.

## Experimental procedures

### Cell culture

K562, CML cell line was procured from NCI and currently stocked at Central Cell line repository at RGCB. LAMA84 and KCL22 were purchased from ATCC (#CRL-3347 and #CRL-3349). All cell lines were *mycoplasma* negative. K562, LAMA84 and KCL22 cells were cultured using RPMI (Himedia #AL162S) with 1% Penicillin/Streptomycin (Invitrogen #15140122), 1% Antimycotic/Antibiotic (GIBCO# 15240-062) and 10% fetal bovine serum (FBS) (Gibco #10270). HEK293 T cells were cultured in standard DMEM (Himedia #AL0078) containing 10% FBS and 1% Penicillin/Streptomycin and 1% Antimycotic/Antibiotic, as described previously (9).

### Cloning

H1.1 was cloned into the EGFPN1 vector using primers listed in Table S1 (Fig. 2*A*). H1.1-EGFPN1 was used for FRAP analysis in K562 cells. For FLIM-FRET experiments, the H2B gene was amplified from the H2B-mCherry construct and subcloned into the EGFPC1 vector to create the H2B-EGFP fusion. H2B-mCherry was a gift from Robert Benezra (Addgene plasmid # 20972; http://n2t.net/addgene:20972; RRID:Addgene_20972) (33). SETDB1 was subcloned from plasmid SETDB1-ORF (34) into EYFPC1 vector (Clonetech; a kind gift from Dr Arumugam Rajavelu, IIT Madras) using primers mentioned in Table S1. pDONR223_SETDB1_WT was a gift from Jesse Boehm & William Hahn & David Root (Addgene plasmid #82291; http://n2t.net/addgene:82291; RRID:Addgene_82291). HIRA was amplified from *HIRA*-YFP plasmid (Kind gift from Dr Almouzni G., CNRS France) and subcloned into Flag-HA-pCDNA3.1 (Addgene #52535). HIRA-FLAG was used to rescue the expression of HIRA in knockdown cells.

### Generation of stable HIRA and SETDB1 knock down in K562 and KCL22 cells

Lentiviral particles expressing shRNA against HIRA and SETDB1 were generated as mentioned in earlier reports (9). 5 x 10^5^ K562 and KCL22 cells were seeded in a six well plate. Lentiviral particles targeting shRNA to *HIRA* gene were produced using HEK293 T cells. *HIRA* shRNA was used in our earlier study (9). Post-infection cells were selected with puromycin (Sigma #P8833) at concentration of (1 μg/ml) for 3 days. Scramble shRNA was used to generate Scramble K562 cells that serves as a control. Post puromycin selection immediately cells were checked for knock-down and it was confirmed by Western blot analysis. *SETDB1* shRNA construct was generated in pLKO.1 vector in a similar manner as mentioned earlier (9). shRNA sequences used in this study has been mentioned in Table S2.

### Ectopic expression of SETDB1

Empty vector EYFPC1 and SETDB1-EYFPC1 were transfected using Lipofectamine 2000 (Invitrogen, #11668019) in K562 cells as per protocol published earlier (9). The transfected cells were screened in presence of 250 μg/ml of G418 (Sigma, #G8168) for 14 days followed by confirmation of overexpression of SETDB1 by Western blot analysis.

### Live cell microscopy and quantitative FRAP analysis

Live cell microscopy and quantitative FRAP analysis were done using a Zeiss LSM980 Airyscan two microscope. The microscope is connected with an accessory incubator that maintains dark conditions as well as a temperature of 37 °C along with 5% CO_2_ levels. For live cell imaging, a glass-bottom dish (Nunc #150680) was used. The glass-bottom dish pre-coated with 0.01% poly-L-lysine (Sigma #P8920) overnight at 37 °C was used for FRAP experiments. Visualization of spatial distribution of H1.1-EGFPN1 in scramble and *HIRA*-shRNA cells was recorded using stack images at 40X apochromat at 5X zoom. Imaging was done using a 60× objective with a 0.95 NA Plan apochromat. The EGFP fluorophore was excited using 488 nm solid-state diode laser lines. Confocal image series were recorded with 8 bit image depth with a frame size of 512 × 512 pixels, a pixel size of 80 nm, and a time interval of 1 s. For photo-bleaching, a ROI of a typical size of 2.5 × 1.5 μm was drawn and used for bleaching. Another ROI of similar size was drawn and used as a reference ROI either from the same cell or from a neighboring cell of similar intensity. Background noise was normalized by using another ROI of similar dimensions and placing it in the background to calculate intensity. Only moderately intense cells were imaged for the FRAP experiment. Photobleaching was done with seven iterations of a 488 nm laser set at 100% transmission. Imaging was done at 0.1% of laser intensity. For every photobleached cell, 3 pre-bleach images and post-bleach frames are taken up to 120 s. Analysis of the intensity calculation was done using ImageJ (https://imagej.net/ij/). To compensate for the movement of the cells because of the bleaching, the StackReg package was used in ImageJ. After calculating the intensity for ROI1 (bleaching), ROI2 (reference), and ROI3 (background), analysis was done using the following mathematical formula. Analysis of the intensity calculation was done using ImageJ. After calculating the intensity for ROI1 (Bleaching), ROI2 (Reference) and ROI3 (Background), analysis was done using following mathematical formula (35).

Formula

(IntensityofROIbeforebleaching−IntensityofROIbackground)/(IntensityofreferenceROIafterbleaching−IntensityofbackgroundROI)

Fluorescence Recovery Curve was plotted with normalized fluorescence intensity against time. Curve fitting was done with one phase association equation for linear regression. Visualization of spatial distribution of H1.1-EGFPN1 in scramble and *HIRA*-shRNA cells was recorded using stack images at 40X apochromat at 5× zoom.

### FLIM-FRET microscopy

K562 cells were co-transfected with H2B-EGFPC1 and H2B-mCherry plasmids using Lipofectamine 2000 (Invitrogen, #11668019) following the manufacturer’s protocol. Briefly, 2 × 10^5^ K562 cells were seeded into a 6-well plate. The following day, the medium was replaced with Opti-MEM (Thermo Fisher Scientific), and cells were incubated at 37 °C. Transfection complexes were prepared by separately diluting plasmid DNA and Lipofectamine 2000 in Opti-MEM, followed by a 5-min incubation at room temperature. The DNA and Lipofectamine mixtures were then combined and incubated for an additional 30 min to allow complex formation. The resulting transfection mixture was added dropwise to the cells. After 5 h, the medium was replaced with RPMI-1640 supplemented with 10% FBS. To generate stable double-positive cell lines, cells were selected with 250 μg/ml G418 (Sigma, #G8168) for 14 days. FACS was subsequently performed to isolate cells expressing both EGFP and mCherry. The resulting stable line, referred to as K562 2FP (two fluorophore-positive) cells, was maintained in RPMI-1640 supplemented with 10% FBS and G418. FLIM-FRET analysis was conducted using live K562 2FP cells on a Leica SP8 confocal microscope equipped with a time-correlated single-photon counting module and a live-cell incubation chamber maintained at 37 °C with 5% CO_2_. Imaging was performed using a 60 × oil immersion objective (1.4 NA, Plan-Apochromat CS2). The donor fluorophore (EGFP) was excited using a 488 nm pulsed laser (femtosecond pulse width, 80 MHz repetition rate). Emission was collected at 550 nm, and the photon count rate was maintained at ∼1000 photons/second.

Fluorescence lifetime data were acquired at a resolution of 512 × 512 pixels or within selected regions of interest. Lifetime measurements were processed using Leica LAS X software, and fluorescence decay was fit using a mono-exponential decay model.

FRET efficiency (E) calculation.

Pixel-wise FRET efficiency (E) was calculated based on the reduction in EGFP fluorescence lifetime in the presence of the acceptor (mCherry) using the equation:

$$E\left(FRET\right)=1-\left(\frac{\tau DA}{\tau D}\right)$$

Where:

τDA is the donor lifetime in the presence of the acceptor (EGFP + mCherry),

τD is the donor lifetime in the absence of the acceptor (EGFP only).

To exclude background and low-signal regions, a photon count threshold was applied. Only pixels above this threshold were included in the analysis. Goodness-of-fit was assessed using the χ^2^ value, which was adjusted to be close to or below one to ensure fitting accuracy. For calculating FRET efficiency ROI was drawn with free hand tool. Bi-exponential fluorescence decay model to fit the decay curve was used to calculate fluorescence lifetime which was used to calculate FRET efficiency.

### Quantification of nuclear fluorescence intensity

Fiji/ImageJ was used to analyze fluorescence images from immunofluorescence and FRAP experiments. Individual nuclei were manually segmented using the freehand selection tool in order to analyze the distribution of H1.1-EGFPN1 in scramble and *HIRA*-shRNA expressed in K562 cells as well as the distribution of H3K9me3. Using the “Make Band” function, a constant-width band that extends inward from the nuclear boundary was created to define the nuclear periphery. The nuclear area that remained after the peripheral band was referred to as the inner nuclear region. Mean fluorescence intensities of the inner nuclear region and the nuclear periphery were calculated from the same nucleus. For each condition, measurements were made for several nuclei. The ratio of inner nuclear to peripheral fluorescence intensity was calculated for each nucleus and used for the detection of intranuclear protein distribution in order to account for variations in overall fluorescence intensity between cells.

### Immunofluorescence

K562 cells were adhered to Poly-L-Lysine (Sigma- P8920) coated frosted slides by using cytospin method. Briefly 5 × 10^5^ cells were adhered on the slide by cytospin at 1000 RPM for 10 min 4% PFA was used for cell fixation. 1× PBS was used for washing the excessive reagents after every step. Permeabilization was done for 10 min using 0.2% tween20 in 1X PBS on ice. Blocking was done at room temperature for 1 hour using 1% BSA. Primary antibodies were diluted in blocking buffer and incubated for overnight at 4 °C. Secondary antibody labelled with fluorophore was diluted using blocking buffer and incubated at room temperature for 1 hour in dark. Hoechst dye was used for nuclear staining. To preserve the photobleaching, suitable mounting medium Antifade (Invitrogen #P36934) was used and coverslip was mounted on the slide. Images were acquired using Nikon A1 Rsi Confocal Laser Scanning Microscope with 60× oil immersion objective. Immunofluorescence images were quantified using ImageJ software (NIH). H3K9me3 fluorescence intensity and its nuclear distribution were analyzed using the “MakeBand” plugin in ImageJ. This approach allows measurement of fluorescence intensity in peripheral and inner nuclear regions. Atleast 30 cells were counted for each condition and data represented as ± SEM.

### Co-immunoprecipitation and LC-MS/MS study

K562 cells were used for pull-down of endogenous HIRA. 10^6^ K562 cells were pelleted and resuspended in NETN buffer (20 mM Tris-HCl, pH7.5, 100 mM NaCl, 1 mM EDTA, 0.1% Nonidet P-40, 1 mM Sodium orthovanadate, 1 mM Sodium fluoride, protease inhibitor cocktail-1tablet) (3, 9). Protein concentration was estimated by Bradford reagent. Isolated protein was further incubated with primary antibody. Further antibody-protein complex was pulled with protein G conjugated Agarose beads (Santa Cruz #SC-2002) and eluted in 2× Laemmli dye. IP samples were checked for protein of interest by Western blot. To identify the interacting partners of HIRA, IP sample were further processed for Mass-spectrometry. For the purposes of proteomic analysis, samples were digested with trypsin overnight at a temperature of 37 °C. The peptides were then concentrated by using a solution of 0.1% formic acid in 80% acetonitrile. The subsequent analysis of peptide samples was done using an Orbitrap Eclipse Tribrid Mass Spectrometer with an UltiMate 3000 RSLCnano UHPLC System or a Vanquish UHPLC System (Thermo Fisher Scientific). The peptides were separated on an RSLC C18 column (100 Å, 2 μm, 75 μm × 50 cm) at 40 °C, with the injection volume being 2 μl. The processing of raw data was done through the use of Proteome Discoverer v3.0.0.757, with the use of the Sequest HT search engine and UniProtKB human database (release 2025_04; taxonomy id: 9606), for the identification of proteins. The static modification was carbamidomethylation of cysteine (+57.021 Da), while the dynamic modifications included methionine oxidation (+15.995 Da) and N-terminal acetylation (+42.011 Da). A maximum of two missed cleavages were allowed. No additional filtering was performed on unique or razor peptides. The resulting data for all proteomics located in the PRIDE database.

### Analysis of IP-Mass spectrometry data

HIRA interacting proteins from LC-MS/MS data were screened by negating the proteins found in IgG control. After eliminating all the common proteins detected in IgG control and HIRA IP samples, one more was deducted from the HIRA IP sample, keratin 4 (a general contaminant). Interacting protein list was further processed for STRING database analysis for human HIRA interactome and for gene ontology (GO) analysis using Database for Annotation Visualization and Integrated Discovery data base. All the proteins detected in Mass spectrometry were together used as a background list. GO analysis was specifically aimed at exploring biological processes. Enrichment significance was calculated based on Fischer’s exact test. GO terms showing *p*-value ≤ 0.05 were considered significant. GO biological processes were visualized using bubble plot where, bubble size represents gene counts and bubble color reflects statistical significance (*p*-value).

### EdU incorporation assay

To trace the replication status upon downregulating HIRA in K562 cells, we employed the click chemistry mechanism by pulsing the cells with 5 μM alkyne-conjugated nucleoside analogue, EdU (5-ethynyl-2′-deoxyuridine) (Thermo Fisher Scientific, #A10044) for 2 h after incubating cells with serum-deprived media (RPMI + 2% FBS) for 16 h, followed by providing the complete medium (RPMI + 10% FBS) for 2 h. This step was followed by coupled fixation and permeabilization with fix buffer A (300 mM sucrose, 0.5% Triton-X-100, and 2% formalin), followed by blocking with 10% FBS-containing PBS. We performed a copper-catalyzed azide coupling reaction that had 10 μM Alexa Fluor 488 azide (Invitrogen, #A10266) in the dark for 1 h at room temperature. Cells were incubated with propidium iodide staining solution after washing with 1X PBST and subjected to FACS analysis using Flow Jo software.

### Apoptosis assay

Around a million K562 cells were collected by centrifugation at 300×*g* and processed according to the manufacturer’s instructions for the Annexin V–FITC Apoptosis Detection Kit (Sigma, #APOAF- 20TST). Briefly, cells were resuspended in binding buffer, followed by the addition of Annexin V–FITC conjugate and propidium iodide. Cells were incubated for 10 min in the dark. Apoptotic populations were analyzed by flow cytometry using a 488-nm laser for Annexin V–FITC detection and a 568-nm laser for PI detection.

### Chromatin immunoprecipitation

Cells (1 × 10^6^) per IP were crosslinked using 1% formaldehyde for 10 min at room temperature followed by addition of glycine (final concentration 0.125 M). Crosslinked cells were sonicated to generate chromatin fragments. ChIP qualified H3.3, H3K9me3, HP1α, EZH2 and H3K27me3 antibodies were used to immuno-precipitate protein-DNA cross-linked fragments. Precipitated complexes were eluted and reverse crosslinked. Enrichment of chromatin fragments was measured by qRT-PCR using SYBR green fluorescence relative to a standard curve of input chromatin. IgG was used as the negative control (3, 9). List of primers have been enlisted in Table S3.

### Protein isolation and immunoblotting

Protein was isolated from freshly pelleted cells using Radioimmunoprecipitation (RIPA) buffer (3, 9) containing using sonication. RIPA buffer contains (10 mM Tris-HCl, pH7.5, 150 Mm NaCl, 5 mM EDTA, 1% Triton X-100, 1% Nonidet P-40, 1% Sodium deoxycholate, 0.1%SDS, 1 mM Sodium orthovanadate, and 1 mM PMSF) (3, 9). Isolated protein concentration was estimated by Bradford Reagent (Bio-Rad #500-0006). By loading equal quantity of proteins on different percentage of gels as per requirements 8%, 10%, 12%, protein was resolved on PAGE followed by transfer it on PVDF membrane (Merck IPVA00010) followed by 1 h blocking with 5% skimmed milk. Antibodies used have been enlisted in Table S4. Visualization of the protein was by enhanced chemiluminescence. Western blot band intensities were quantified using ImageJ software (NIH). ROIs were drawn around individual bands and the intensity was measured following background subtraction. The intensity of each target protein band was normalized to the respective GAPDH band intensity for whole-cell lysates. For histone modification analysis, band intensities were normalized to total histone H3 levels. Quantification was performed from at least three independent biological experiments and data are presented as mean ± SEM.

### Quantitative RT-PCR

RNA was isolated using RNAeasy kit (Qiagen #74106) following the manufacturers guidelines. cDNA synthesis was aided by random hexamer along with Reverse transcriptase enzyme (ABI Biosystems #4368814). qRT-PCR was performed with SYBR-Green (ABI Biosystems #4309155) and analyzed by using relative standard curve method (3, 9). Primers used have been listed in Table S5.

### Statistical analysis

Statistical analysis was performed using GraphPad Prism (https://www.graphpad.com/features) version 9 (GraphPad software). Data have been represented as a mean ± SEM (standard error of the mean). For Western blot, qRT-PCR, immunofluorescence, FRAP, and FLIM-FRET analyses, at least three independent biological replicates were considered. Statistical tests used for comparing two groups are the Student's *t* test, and for multiple test groups, two-way ANOVA has been used. If any other statistical tests have been used, it has been mentioned in the respective figure legend.

## Ethics approval and consent to participate

Not applicable.

## Consent for publication

Not applicable.

## Data availability

All data have been submitted along with the manuscript.

## Supporting information

This article contains supporting information.

## Conflict of interest

The authors declare that they have no conflicts of interest with the contents of this article.

## Supporting information

Table S8

**Abbreviations**
BCR-ABL, Breakpoint Cluster Region protein- Abelson Tyrosine-Protein Kinase 1
CML, chronic myeloid leukemia
EZH2, enzyme unit Enhancer of Zeste Homolog 2
FACS, fluorescence-activated cell sorting
FBS, fetal bovine serum
FLIM-FRET, fluorescence lifetime imaging microscopy-forster resonance energy transfer
FRAP, Fluorescence Recovery After Photobleaching
GO, gene ontology
HIRA, histone cell cycle regulator A
PEP, posterior error probability
ROI, Region of Interest
SETDB1, SET Domain Bifurcated Histone Lysine Methyltransferase 1
TK, tyrosine kinase
