# Supplementary material for: HIRA-SETDB1-H3K9me3 axis regulates chromatin architecture in leukemia cells
Source: J Biol Chem. 2026 Apr 27;302(6):113081. doi: 10.1016/j.jbc.2026.113081 (PMC13226233; doi:10.1016/j.jbc.2026.113081)
Supplement: Supplementary Information [file mmc1.pdf]

HIRA-SETDB1-H3K9me3 axis regulates chromatin architecture in leukemia cells

Mayur Balkrishna Shirude[1,2], Anjali Devarajan[1], Sai Adarsh Sahu[1, 3], Ananda Mukherjee[4], Debasree Dutta[1]*

[1]Rajiv Gandhi Centre for Biotechnology, Regenerative Biology Program, Thycaud PO, Poojappura, Thiruvananthapuram 695014, Kerala, India

[2]Manipal Academy of Higher Education, Manipal, Karnataka State, 576104, India.

[3]Regional Centre for Biotechnology, Faridabad, Haryana, 121001, India

[4]Cancer Biology Laboratory, Department of Medical Oncology, Sri Ramachandra Institute of Higher Education and Research, Porur, Chennai, 600116, India

* Correspondence to debasreedutta@rgcb.res.in

Supplementary Information

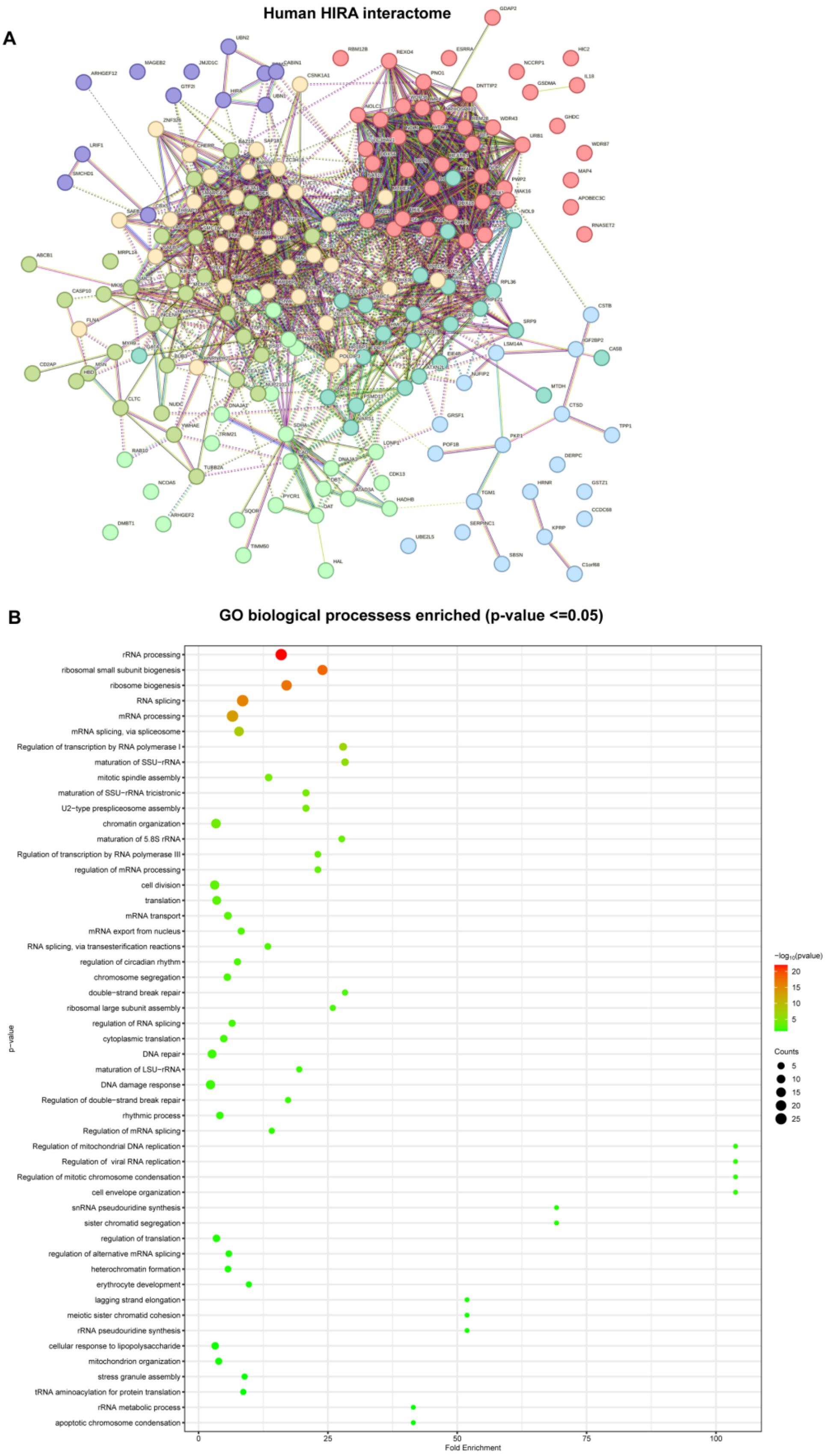

Supplementary Figure S1. Top panel represent the interactome of HIRA in K562 cell. Lower panel represent the two major clusters which are part of the interactome. B.

Bubble graph demonstrate the Gene ontology (GO) analysis by DAVID bioinformatics tool (https://david.ncifcrf.gov) for the biological processes (BP), with a significant p-value, for HIRA deduced from LC-MS/MS study.

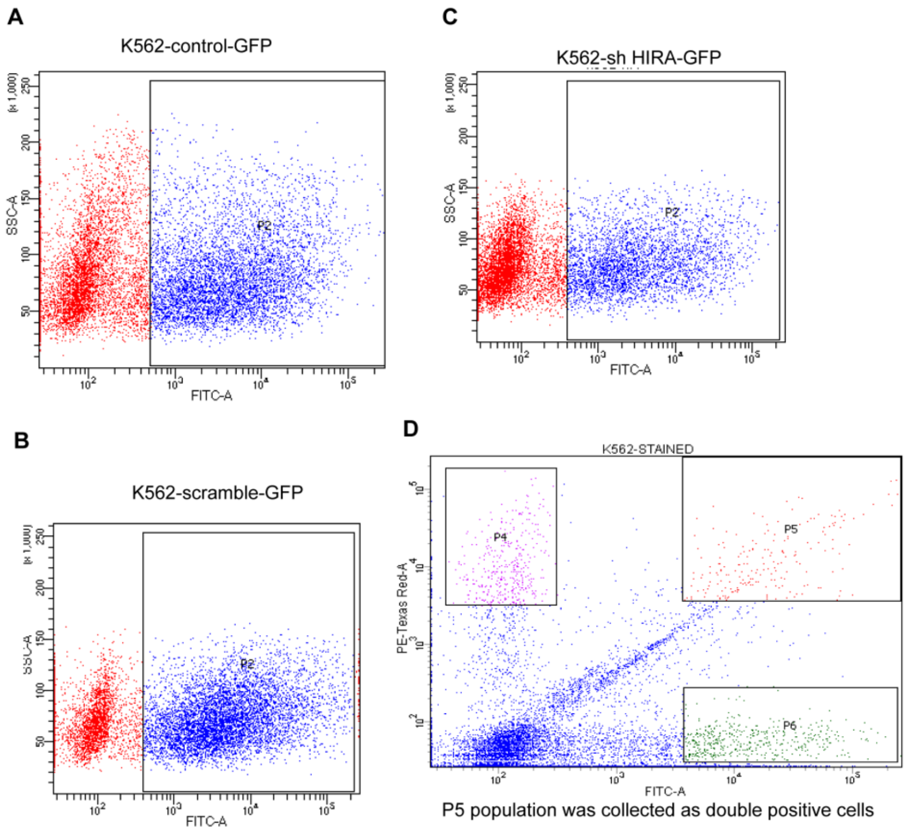

Supplementary Figure S2. A, B, C. FACS of H1.1-EGFP positive control, scramble-shRNA and HIRA-shRNA expressing K562 cells. P2 represent the sorted population used for FRAP analysis. D. FACS for the H2B-mCherry/EGFP expressing K562 cells. P5 population was used for further experiments.

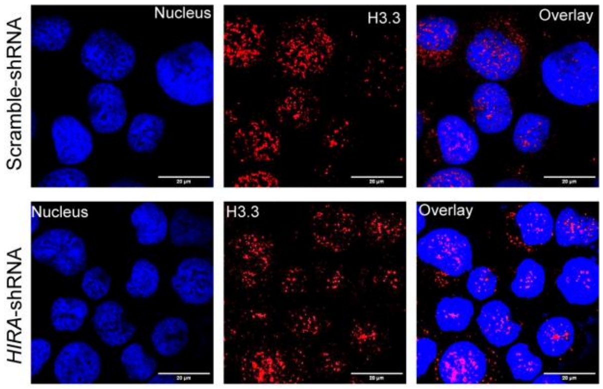

Supplementary Figure S3. Immunofluorescence analysis for the expression of histone H3.3 in K562 cells expressing scramble or *HIRA*-shRNA.

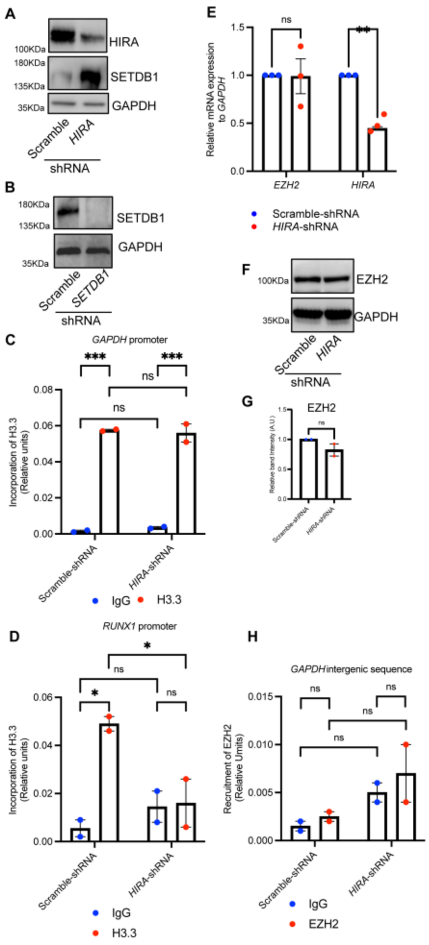

Supplementary Figure S4. A. Western blot analysis for the expression of HIRA and SETDB1 upon downregulation of HIRA in K562 cells. The blot image of Fig. 8B HIRA has been used as the samples are same. B. Confirmation of SETDB1 knockdown by Western blot analysis for the expression of SETDB1 in K562 cells. C, D. Quantitative

ChIP analysis for the enrichment of H3.3 within *GAPDH* promoter and *RUNX1* promoter. IgG is the negative control. A two-way ANOVA statistical analysis was done followed by Šídák's multiple comparisons tests (mean of columns, mean of rows), ***p<0.001, *p<0.05, ns=not significant, N=2 biological replicates. E. Quantitative RTPCR analysis for the expression of EZH2 and HIRA in K562 cells. A two-way ANOVA statistical analysis was done followed by Šídák's multiple comparisons tests, **p<0.01, ns=not significant, N=3 biological replicates. F. Western blot analysis for the expression of EZH2 in K562 cells. G. Bar graph represents the band intensity EZH2 normalized to GAPDH in the K562 cells. Unpaired t-test was performed for the statistical analysis, ns=not significant, N=2. H. Quantitative ChIP analysis for the recruitment of EZH2 within *GAPDH* intergenic sequence. IgG is the negative control. A two-way ANOVA statistical analysis was done followed by Šídák's multiple comparisons tests (mean of columns, mean of rows), ns=not significant, N=2 biological replicates.

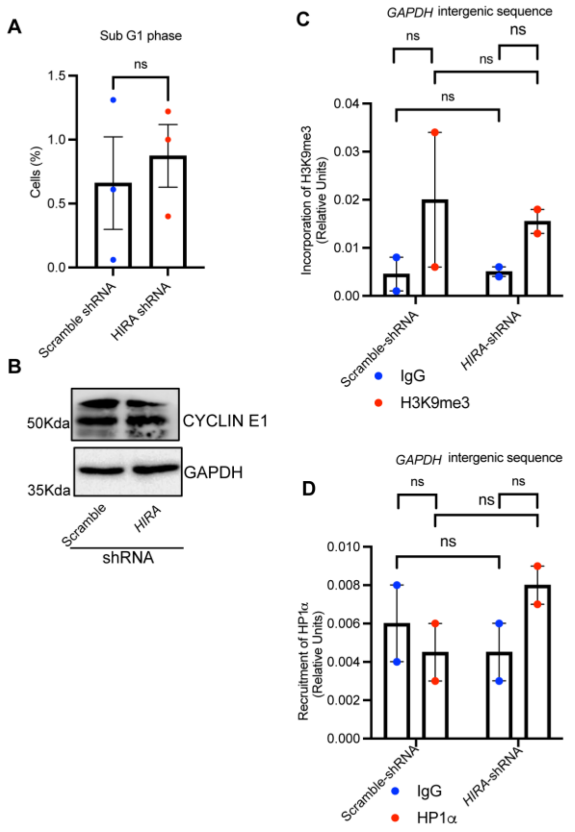

Supplementary Figure S5

Supplementary Figure S5. A. Bar graph demonstrates the fraction of sub G1 population in scramble and *HIRA*-shRNA expressing cells analyzed in Fig. 7A. Unpaired t-test was performed for the statistical analysis, ns=not significant, N=3. B. Western blot analysis for the expression of Cyclin E1 in cells analyzed in A. Fig. 8B GAPDH image has been used as the same blot was stripped and probed for Cyclin E1. C. Quantitative ChIP analysis for the incorporation of H3K9me3 within GAPDH promoter. IgG is the negative control. A two-way ANOVA statistical analysis was done followed by Šídák's multiple comparisons tests, ns=not significant, N=2 biological replicates. D. Quantitative ChIP analysis for the recruitment of HP1α within the GAPDH promoter. IgG is the negative control. A two-way ANOVA statistical analysis was done followed by Šídák's multiple comparisons tests, ns=not significant, N=2 biological replicates.

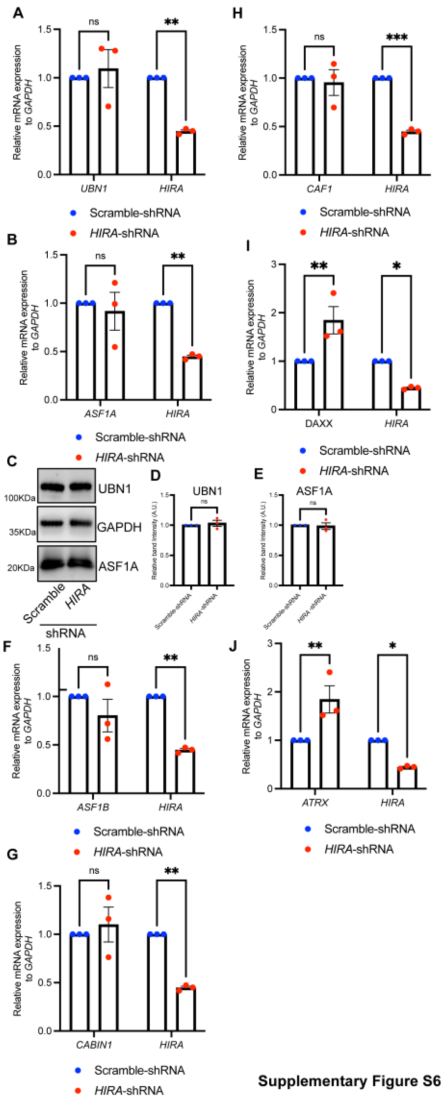

Supplementary Figure S6

Supplementary Figure S6. A, B. Quantitative RTPCR analysis for the expression of UBN1 and ASF1A in K562 cells. A two-way ANOVA statistical analysis was done followed by Šídák's multiple comparisons tests, **p<0.01, ns=not significant, N=3 biological replicates. C. Western blot analysis for the expression of UBN1 and ASF1A in K562 cells. D, E. Bar graph represents the band intensity of UBN1 and ASF1A normalized to GAPDH in the K562 cells. Unpaired t-test was performed for the statistical analysis, ns=not significant, N=3. F-J. Quantitative RTPCR analysis for the expression of ASF1B, CABIN1, CAF1, DAXX and ATRX in K562 cells. A two-way ANOVA statistical analysis was done followed by Šídák's multiple comparisons tests, *p<0.05, **p<0.01, ns=not significant, N=3 biological replicates.

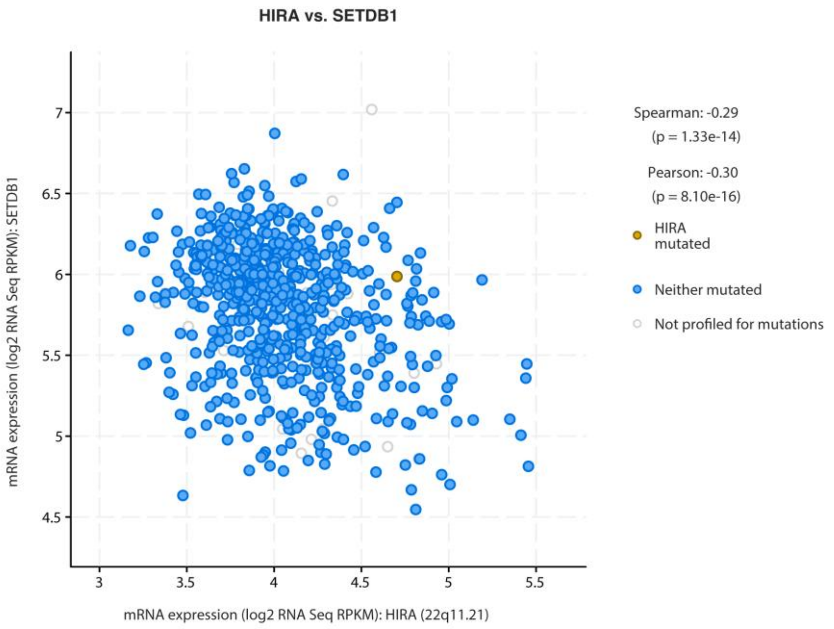

Supplementary Figure S7. Scatter plot for the co-expression of HIRA with SETDB1 in 671 AML patient samples from TCGA database analysed by cBioPortal.

Supplementary video 1 and 2. Z-stack image for the expression of H1.1 EGFP in K562 cells expressing scramble-shRNA and *HIRA*-shRNA respectively.

Supplementary Tables associated with materials and methods

Table S1. Cloning Primers

H1.1_EGFPN1 F	CCAAGCTTACCGCCATGTCTGAAACAGTGCCTCCCGC
H1.1-EGFPN1_R	CGGGATCCCCCTTTTTCTTGGGTGCCGC
H2B-EGFPC1_F	CCCTCGAGATGCCTGAACCCTCTAAGT
H2B_EGFPC1_R	GCGTCGACAGAGCTAGTGTACTTGG
HIRA_FLAG_F	CTCGAGATGAAGCTCCTGAAGCCGAC
HIRA_FLAG_R	GAATTCCTACTTGTCCCTCAGGATGTC
SETDB1_pEGFPC1_F	CGCTCGAGGTATGCCAACTTTGTACAAA
SETDB1_pEGFPC1_R	CGGTCGACTCAGAGATTTTGAGACAC

Table S2. ShRNA used in this study

shRNA_SETDB1_F	CCGGGCTCAGATGATAACTTCTGTACTCGAGTACAGAAGTTATCATCTGAGC TTTTTG
shRNA_SETDB1_R	AATTCAAAAAGCTCAGATGATAACTTCTGTACTCGAGTACAGAAGTTATCATC TGAGC
shRNA_HIRA_F	CCGGCTCTATCCTCCGGAATCATTCCTCGAGGAATGATTCCGGAGGATAGAG TTTTTG
shRNA_HIRA_R	AATTCAAAAACTCTATCCTCCGGAATCATTCCTCGAGGAATGATTCCGGAGG ATAGAG

Table S3. ChIP primers used in this study

Gene name	Forward	Reverse
Ki67 promoter	CACGAGACGCCTGGTTACTA	TGAGGCTCAGGGGAAAGTG
PCNA promoter	CGCTCTGAGGCTCCTGAAG	GCGGGAAGGAGGAAAGTCTA
SETDB1 promoter	CTGCCAGTCTCTTCTCACGT	CAGAGGCGACGAAAACTAAGG
BCR-ABL up1	GGAAGGGGATGAGAAGGTCC	CTTGCTAACCTCCTGCCATC
BCR-ABL up2	CCTCACATGCAAAATGGGCT	GACCTTCTCATCCCCTTCCC
HP1α promoter	TGAGAACACGTGAAATGGCG	GACAAACTCCCGCCAACAC
GAPDH promoter	CCACATCGCTCAGACACCAT	CCCGCAAGGCTCGTAGAC
GAPDH intergenic sequence	CCTGGCCTCTCACACTCA	AGAACCCTTGCTCTCCAC
RUNX1 promoter	AGCTGTTTTCAGGGTCCTTACTC	GGGGATACGCATCACAACA

Table S4. Primary Antibody Used in this study

Primary antibody	Catalog number	Company	Dilutions
HIRA	04-1488	Merck Millipore	1-1000 (WB)
GFP	2956S	Cell Signaling Technology	1-1000 (WB)
Ki67	MA5-14520	Thermo-Fisher Scientific	1:2000 (WB)
H3	9715S	Cell Signaling Technology	1-2000 (WB)
PCNA	sc7907	Santacruz	1:3000 (WB)
H3K9me3	ab8898	Abcam	1:2000 (WB)<br>1:200 (IF)<br>1.5μg per IP
H3K27me3	ab6002	Abcam	1:2000 (WB)<br>1.5μg per IP
H3K4me1	ab8895	Abcam	1:2000 (WB)
H3K4me2	ab32356	Abcam	1:2000 (WB)
H3K4me3	ab8580	Abcam	1:2000 (WB)
H3K36me3	ab9050	Abcam	1:2000 (WB)

H3K27ac	ab4729	Abcam	1:2000 (WB)
H3K9ac	ab10812	Abcam	1:2000 (WB)
H3.3	ab176840	Abcam	1:2000 (WB)<br>1:200 (IF)<br>1.5μg per IP
GAPDH	G9545	Sigma	1:10000 (WB)
SETDB1	GTX115305	Genetex	1:3000 (WB)
BCR-ABL	ab187831	Abcam	1:2000 (WB)
Cleaved PARP1	5625	Cell Signaling Technology	1:1000 (WB)
Total PARP1	436400	Thermo Scientific	1:1000 (WB)
BAX	B8429	Sigma	1:1000 (WB)
Ki67	sc23900	Santacruz	1:2000 (WB)
EZH2	ab3748	Abcam	1:2000 (WB)<br>1.5μg per IP
HP1α	ab109028	Abcam	1:2000 (WB)<br>1.5μg per IP
ASF1A	2990S	Cell Signaling Technology	1:2000 (WB)
UBN1	sc515340	Santacruz	1:600 (WB)
CYCLIN E1	4129	Cell Signaling Technology	1:1000 (WB)

Secondary antibodies used in the study

Antibody	Company	Catalog number	Dilution
Goat anti mouse IgG-HRP	Santacruz	SC2005	1-2000 (WB)
Goat anti-rabit alexa-fluor 488	Invitrogen	A11008	1-200 (IF)
Goat-anti-mouse alexa fluor 568	Invitrogen	A11004	1-200 (IF)

Table S5. qRT-PCR primers used in this study

Gene name	Forward primer	Reverse primer
GAPDH	CACCAGGGCTGCTTTTAACTCTGGTA	CCTTGACGGTGCCATGGAATTTGC
KDM4B	AGACGTATGATGACATCGACGA	CGTAGATCGGGGAGACAAAGG
KDM4A	GAAGCCACGAGCATCCTATGA	GCGGAACTCTCGAACAGTCA
KDM4C	CGAGGTGGAAAGTCCTCTGAA	GGGCTCCTTTAGACTCCATGTAT
KDM4D	ATCGCCATTTGGCAAACAGTA	GGGTGTATTGACGCCTTCTATGA
KDM4E	AATCACGGCTTCAACTGCG	CATAACTCTCGGGTTGCACAA
SUV39H2	TACTCGTCTTCCCCGAATAGC	GGCTGTGGTCAATAGAATCTGAA
SUV39H1	CATCTGGGACGCATCACTGTA	TCACCAACACGGTACTCATTG
SETDB1	TAAGACTTGGCACAAAGGCAC	TCCCCGACAGTAGACTCTTTC
BCR-ABL	TGTGAAACTCCAGACTGTCCACA	AAAGTCAGATGCTACTGGCCG
CBX5	GACAGGCGCATGGTTAAGG	CCTGGGCTTATTGTTTTCACCC
ATRX	TGAAACTGTAAATGAAGATGCGT	CTTTGTCTTCATTCAGCACTGG
EZH2	ATGGGCCAGACTGGGAAGA	CTTCCGCCAACAAACTGG
ASF1B	TCATCCGAGTGGGCTACCTACG	GTTGTTGTCCCAGTTGATATGGA
DAXX	GATACCTTCCCTGACTATGGGG	GTAACCTGATGCCCACATCTC
ASF1A	TGGCATCTAATCCCAGGGTCA	TTCTGATGTGGACCATCCCTT
UBN1	CCTGAATCCTGCGTTTTTGAAG	GCAGCGTTTGTGATCTGGTT
CABIN1	AGGATTGATTTGTCGGACTACCA	TAGCCACAGAGGTGTAGGAGA
CAF1	TTAGACCGAAACTTGTCAACGG	GTCTGGCTGCTCATTCGAGT

Supplementary Tables associated with results section

Supplementary Table S6 Excel sheet: HIRA IP interaction partners

Supplementary Table S7 Excel sheet: IgG sample

Supplementary Table S8 Excel sheet: DAVID chart GO: Biological Process (sheet 1), Uniprot Accession numbers of protein analysed as interaction partners of HIRA.
